# Catecholaminergic nucleus integrity and Alzheimer's pathology, symptoms, and progression

**DOI:** 10.1002/alz.70749

**Published:** 2025-09-29

**Authors:** Michael C. B. David, Magdalena A. Kolanko, Thomas D. Parker, Ramin Nilforooshan, Karl A. Zimmerman, Cristina Bonet Olivares, Karen Hoang, Johanna Brandt, Charikleia Triantafyllou, Peter J. Lally, Gregory Scott, CR&T Group of UKDRI, David J. Sharp, Paresh A. Malhotra

**Affiliations:** ^1^ UK Dementia Research Institute Care Research and Technology Centre Imperial College London London UK; ^2^ Imperial College London, Brain Sciences, South Kensington London UK; ^3^ The Dementia Research Centre, Department of Neurodegenerative Disease University College London London UK; ^4^ Surrey and Borders Partnership NHS Foundation Trust Leatherhead Surrey UK

**Keywords:** catecholamines, cognition, disease progression, dopamine, locus coeruleus, neuromelanin, neuropsychiatric symptoms, noradrenaline, norepinephrine, substantia nigra

## Abstract

**BACKGROUND:**

The noradrenergic locus coeruleus (LC) accumulates pathology early in Alzheimer's disease (AD), with LC dysfunction contributing to symptoms and disease progression. We investigated LC and substantia nigra (SN) integrity in healthy controls and AD participants.

**METHODS:**

Ninety‐three AD participants and 29 controls underwent neuromelanin magnetic resonance imaging. LC and SN contrast, reflecting nucleus integrity, related to cognitive and neuropsychiatric symptoms, as well as cognitive decline and atrophy rates.

**RESULTS:**

LC – but not SN – integrity was reduced in AD versus controls (*b *= −0.39, *p* = 0.001) and within AD was associated with global cognition (*b *= 8.53, *p* = 0.04) and neuropsychiatric symptoms, accounting for SN. An AD subgroup with reduced SN integrity had worse cognition. LC integrity predicted plasma phosphorylated tau protein 217 (*b *= −0.30, *p* = 0.03). Lower LC and SN integrities were both related to faster cognitive decline (LC: *b *= −4.74, *p* = 0.048; SN: *b *= −2.27, *p* = 0.03), accounting for one another.

**DISCUSSION:**

Catecholaminergic nucleus integrity plays an important role in AD. Both systems are relevant to cognitive performance and decline. LC, in particular, relates closely to symptoms, pathology, and rate of progression.

**Highlights:**

- In symptomatic AD, LC integrity reflects cortical AD pathology, measured by pTau217.
- LC integrity predicts cognitive function in AD, independent of cortical atrophy.
- LC and SN integrity independently relate to attentional performance.
- Symptoms of anxiety, depression, and apathy are associated with lower LC integrity.
- LC and SN relate to cognitive decline rate and left LC predicts atrophy rate.

## 1 BACKGROUND

Wide‐reaching efferents from the pontine locus coeruleus (LC) supply much of the cortex with noradrenaline (or norepinephrine)^1^ – a key neurotransmitter for cognition and behavior.^2^, ^3^, ^4^ In Alzheimer's disease (AD), the LC accumulates toxic hyperphosphorylated tau decades before symptoms, leading to neuronal death, reduced integrity,^5^ and symptoms.^2^, ^6^

**FIGURE 1 alz70749-fig-0001:**
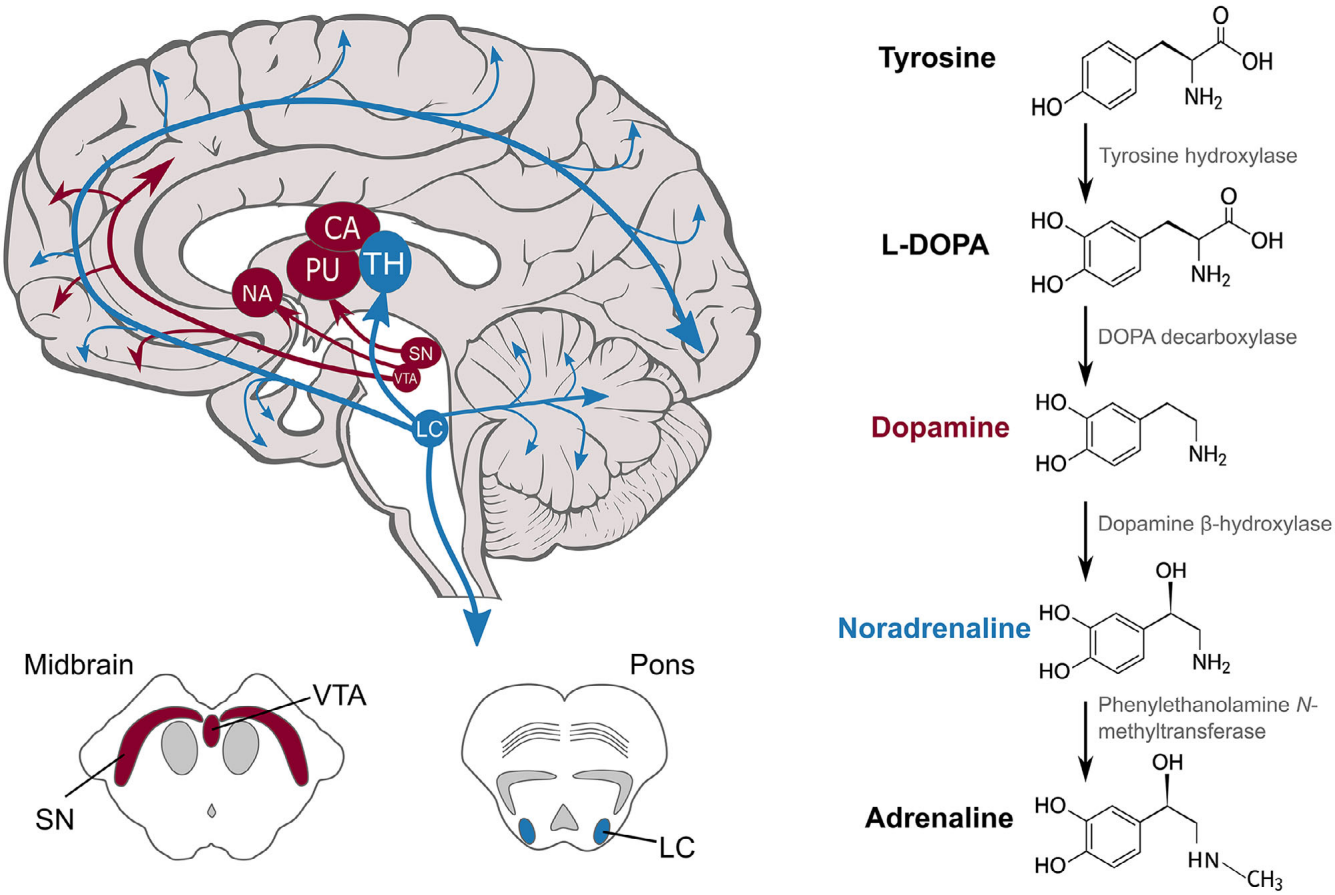
Sources and biosynthesis of catecholamines. Top left: location and projections of LC (blue) and SN (red). Bottom left: anatomy of nuclei on axial slices through brainstem. Right: biosynthetic pathway of dopamine, noradrenaline, and adrenaline. CA, caudate; LC, locus coeruleus; NA, nucleus accumbens; PU, putamen; SN, substantia nigra; TH, thalamus; VTA, ventral tegmental area.

The midbrain substantia nigra (SN), the main source of central dopamine (Figure 1), is a key pathological locus in Parkinson's disease (PD), but has received less attention in AD. However, Lewy bodies, plus tau aggregates and neuronal loss, have been reported in the SN in AD.^7^, ^8^, ^9^ Additionally, *post mortem* studies and positron emission tomography (PET) imaging have demonstrated reduced dopaminergic function in AD.^10^, ^11^ This is recognized in recent diagnostic criteria, which include α‐synuclein as a common non‐AD co‐pathology.^12^

Owing to its small size and deep location, the LC has been challenging to image in vivo with conventional techniques. The development of neuromelanin‐sensitive magnetic resonance imaging (MRI) represents a significant breakthrough. Neuromelanin accumulates over the lifespan in the LC and SN, appearing bright on targeted sequences, allowing accurate segmentation.^13^ Importantly, neuromelanin undergoes microglial clearance following cell death, causing signal reduction in neurodegenerative conditions.^14^ Reduced LC contrast has been repeatedly demonstrated in AD,^15^, ^16^, ^17^ as have SN reductions in PD^18^ but not AD.

LC contrast relates to cognition, particularly memory, in older healthy people,^19^ those with mild cognitive impairment (MCI),^20^ and AD‐mutation carriers.^21^ However, there has been only limited work investigating the relationship between LC contrast and cognitive subdomains in sporadic clinical AD.^15^, ^22^, ^23^ Neuromelanin MRI can quantify integrity in both LC and SN, allowing exploration of their shared and distinct associations with cognition.^19^

Catecholaminergic control of arousal is also relevant to neuropsychiatric symptomatology.^4^, ^24^ Importantly, neuropsychiatric symptoms are inadequately addressed by existing therapies.^25^ Most notably, anxiety, depression, and apathy are closely associated with arousal and noradrenergic tone (see Brunello et al.^4^ for review). In one study of AD, lower LC contrast predicted worse neuropsychiatric symptoms, though without accounting for any contribution from the SN.^26^

LC integrity in AD appears to reflect overall disease state. Reduced LC contrast predicts greater amyloid beta (Aβ) burden and relates to cortical tau more strongly than Aβ PET or hippocampal volume do.^17^, ^27^, ^28^, ^29^ Recent developments in plasma biomarkers, particularly phosphorylated tau protein 217 (pTau217), offer practical in vivo measures of AD pathology and are strongly associated with amyloid and tau PET tracer uptake.^30^ Neurofilament light (NfL) and glial fibrillary acidic protein (GFAP) – non‐specific markers of neurodegeneration and astrocyte activation, respectively – are also measurable in plasma.^31^, ^32^ Previous work has only explored the relationship between LC contrast and these biomarkers in healthy older adults.^33^, ^34^

LC integrity may also influence the trajectory of AD.^27^, ^35^ There is considerable variability in AD progression rate. Noradrenaline has substantial neuroprotective effects,^35^ including reducing inflammation and excitotoxicity,^36^ and stimulating Aβ clearance by microglia.^36^ In AD mouse models, LC damage promotes neuroinflammation, Aβ, and tau pathology.^37^, ^38^ Accordingly, across assessment of multiple brainstem nuclei, LC neuronal density was most associated with the rate of age‐related cognitive decline.^39^ Lower LC contrast predicts conversion to MCI/AD^23^, ^28^ and also a steeper slope of memory decline in mild dementia.^27^ Similarly, dopamine enhances Aβ degradation in mice and therefore dysfunction may exacerbate disease progression.^40^ Also, AD patients with parkinsonism show faster cognitive decline.^41^ However, the relationship of LC and SN integrity to disease progression has not been examined at later disease stages.

We systematically evaluated relationships between catecholamine nucleus integrity, cognitive and neuropsychiatric symptoms, rate of decline, and relevant plasma biomarkers in a cohort of 93 participants with AD and 29 healthy older people. We specifically tested the following hypotheses: (1) LC integrity and SN integrity are independently associated with cognition; (2) lower LC integrity is related to arousal‐related neuropsychiatric symptoms; (3) lower LC integrity correlates with higher plasma pTau217, GFAP, and NFL levels; and (4) LC integrity and SN integrity predict the rate of disease progression measured using longitudinal cognitive testing and atrophy rate.

## 2 METHODS

### 2.1 Participants

AD participants (*N* = 93) were recruited as part of two studies: (1) “Minder,” an ongoing longitudinal community‐based dementia cohort study run by the Care, Research and Technology Centre of the United Kingdom Dementia Research Institute (UK DRI) (*N* = 38);^42^ and (2) “Physiological Correlates of Noradrenergic Add‐on Therapy” (PCNorAD), an experimental medicine add‐on study to the NorAD clinical trial (*N* = 55).^43^ Participants recruited from NorAD underwent the procedures for this study at least 1 month after completing participation in the trial. Twenty‐nine age‐matched healthy controls (HC) were also recruited as part of PCNorAD.

All AD participants had a pre‐existing clinical diagnosis of AD that was reviewed in a multidisciplinary team meeting, composed of neurologists, psychiatrists, and neuroradiologists, as part of this study. Diagnosis was confirmed upon review of available clinical and research neuroimaging as well as other investigations including cerebrospinal fluid (CSF) and PET biomarkers. HC were recruited if >60 years old and with no symptoms of cognitive impairment. All participants were free of major neurological illnesses other than AD.

**RESEARCH IN CONTEXT**

1. **Systematic review**: PubMed and GoogleScholar literature searches revealed evidence for LC pathology early in AD. The LC's relevance to cognition and progression to AD has mostly been reported in healthy or prodromal cohorts. There is limited work exploring SN integrity in AD. We extended pre‐existing work, examining both nuclei in patients across a range of AD severity.
2. **Interpretation**: We demonstrate LC integrity significantly contributes to cognitive and neuropsychiatric symptoms in AD, independently of SN integrity. LC integrity is associated with pathological burden and disease progression, although the integrity of both nuclei relates to attention and cognitive decline rate, independently of each other. Together, these results highlight the influence of noradrenergic function, in particular, on symptoms and disease trajectory.
3. **Future directions**: Therapeutic approaches that directly modulate noradrenergic tone and/or protect the integrity of the LC have the potential for symptomatic and possibly disease‐modifying benefit.

### 2.2 Ethical approval

The Minder and PCNorAD studies were[Fig alz70749-fig-0001] approved by the London‐Surrey Borders Research Ethics Committee (19/LO/0102) and London‐Central Research Ethics Committee (18/LO/0249), respectively. All participants with the capacity to consent provided written informed consent for participation and for their data to be included. Those without capacity were enrolled in accordance with the Mental Capacity Act (2005) on recommendation of an assigned consultee.

### 2.3 MRI acquisition

MRI scans were acquired once for all controls and for AD participants in PCNorAD and approximately annually for AD participants in Minder. For some participants, MRI data from different visits were used for different analyses to meet the specific criteria for each analysis (see below). MRI data were obtained using a Siemens Verio 3T MRI scanner with a 32‐channel head coil. Participants underwent a T1‐weighted three‐dimensional magnetization‐prepared rapid acquisition gradient‐echo (3D‐MPRAGE) sequence.

The parameters were as follows: flip angle = 9°, echo time (TE) = 2.98 ms, repetition time (TR) = 2300 ms, inversion time = 900 ms, bandwidth = 240 Hz/pixel, acquisition matrix = 256 × 240 × 160, voxel size = 1.0 × 1.0 × 1.0 mm, and GRAPPA acceleration factor = 2. LC and SN contrast was quantified using the following MRI sequence (3D T2*‐weighted multi‐echo gradient‐echo with a magnetization transfer preparation pulse), which was aligned perpendicularly to the plane of the participant's brainstem based on the sagittal reconstruction from the MPRAGE sequence and used the following parameters: flip angle = 20°; TE = 7.5, 15.0, and 22.5 ms; TR = 62 ms; acquisition matrix = 384 × 384 × 48; voxel size = 0.67 × 0.67 × 1.34 mm; bandwidth = 230 Hz/pixel; GRAPPA acceleration factor = 2; and slice partial Fourier factor = 6/8. The first echo time was analyzed.

### 2.4 MRI analysis

#### 2.4.1 Neuromelanin contrast

Images were bias corrected using N4BiasFieldCorrection by Advanced Normalization Tools version 2.1. LC contrast was quantified as in David et al.^44^ (Figure S1). Briefly, the LC was manually identified as two hyperintense areas in the pons around the base of the fourth ventricle.^45^ Contrast values were calculated as a ratio between a five‐voxel region on both the left and right against a 7 × 7 voxel square reference region in the nearby pons, using the following equation:^45^

$$\begin{array}{c} (\mathrm{Mean}\;\mathrm{of}\;\mathrm{left}\;\mathrm{and}\;\mathrm{right}\;\mathrm{LC}\;\mathrm{contrast}-\;\mathrm{Mean}\;\mathrm{reference}\;\mathrm{contrast})/\\ \mathrm{Standard}\;\mathrm{deviation}\;\mathrm{of}\;\mathrm{reference}\;\mathrm{contrast} \end{array}$$

This was conducted across five consecutive slices and then averaged to give a single value per side, and the average of the two sides was calculated. We used two independent raters for each scan with an average taken. The ICC between the two raters was very high (0.88 to 0.96). Where there was a large disagreement regarding contrast ratio between raters (>0.45), scans were re‐examined, and a consensus was reached.

While a manual method was employed for quantification of the LC to prevent the risk of misplacement of regions of interest (ROIs), a semi‐automated method was employed for the quantification of SN contrast (Figure S2), owing to its larger size and the existence of established ROI masks.^46^ In addition to these SN region masks, a circular reference region four voxels in diameter and spanning seven consecutive slices in the nearby cerebral peduncle on the left and right, was created. First, a Montreal Neurological Institute (MNI) template image and the bias corrected neuromelanin‐sensitive brainstem slab images were registered to each participant's T1 image using the default antsRegistrationSyNQuick.sh process of rigid, affine, and non‐linear transformations.^47^ Then the transforms from the MNI to T1 registrations were applied to the SN and reference ROIs, after which, the inverse of the transforms from the brainstem slab to T1 registrations was applied to the ROIs to move them to the participant space. The mean and standard deviation of the contrast in the ROIs for each participant was then extracted. Contrast ratios were calculated for the left and right SN region relative to the reference regions using the same formula as for the LC. Ratios were then averaged across sides to give a single SN contrast ratio.

We also produced a composite metric of LC and SN contrast by dividing each participant's value by the median value in the control group, for LC and SN separately. Given that they are collected using the same sequence, if proved useful, this metric could be easily computed in future clinical and scientific work. These standardized values were then summed to give a “LCSN” value representative of the combined contrast in both nuclei.

#### 2.4.2 Gray matter volume and atrophy

All T1 images underwent brain extraction using HD‐BET^48^ and tissue segmentation using FMRIB’s Automated Segmentation Tool^49^ to give total gray matter volume and estimated total intracranial volume. Then, for subjects with multiple scans, an established pipeline was used for longitudinal analysis in SPM12 (University College London).^50^ Briefly, T1 images at baseline and follow‐up were segmented into gray matter, white matter, and CSF. Where two follow‐up scans were available, the later of the two was used to maximize reliability of the atrophy rate measure. The segmentations were visually quality checked. Each participant's baseline T1 was iteratively co‐registered to their follow‐up image to produce a midpoint “participant average‐space” image, which was also then segmented into the three tissues. The gray matter segmentation was binarized to produce a gray matter specific mask, which was then divided into left and right by the midline. For each participant a three‐dimensional voxel‐wise “Jacobian determinant” (JD) map was produced. The JD measures how much a volume changes during a transformation and is applied to serial brain imaging data to show the change in volume between scans as a measure of atrophy rate.^50^ The bilateral, left, and right gray matter masks for each participant were then applied to the JD map. These values were multiplied by 100 to produce percentage change per year.

### 2.5 Neuropsychological assessment

For the analysis of rate of cognitive decline, the Alzheimer's Disease Assessment Scale–Cognitive Subscale (ADAS‐Cog) 14^51^ was used. This is frequently used in assessing changes in cognition over time in AD, particularly in clinical trials. For AD participants in the Minder study, this was performed routinely every 6 months. For PCNorAD participants, this was performed once at the start of the NorAD trial (before starting either drug or placebo) and in a subgroup of participants, at one further time point at least 3 months after the trial. This gave a range of 2 to 7 ADAS‐Cog scores per participant included in this analysis. If multiple scans were available for a given participant, the scan closest to the first ADAS‐Cog was used in the cognitive decline rate analysis. For all participants, this scan fell during, or within 1 year of, the period of longitudinal cognitive testing.

The Addenbrookes Cognitive Examination‐III (ACE)^52^ was selected as the cross‐sectional measure on the day of MRI scanning as it is quicker and more practical to administer than the ADAS‐Cog. It has the added advantage of providing five cognitive subdomain scores (attention, language, memory, visuospatial ability, and fluency) that can be independently related to imaging markers. By not using the ADAS‐Cog on the day of the MRI scan, it also ensured that Minder participants did not have multiple ADAS‐Cog assessments within a 6‐month period, which would increase the risk of learning effects. If multiple contemporaneous MRI‐ACE timepoints were available, the first was used.

### 2.6 Neuropsychiatric assessment

The Neuropsychiatric Inventory (NPI)^53^ was used to assess neuropsychiatric symptoms for the AD participants. Total scores (frequency × severity) were collected across 12 domains: delusions, hallucinations, agitation, depression, anxiety, elation, apathy, disinhibition, irritability, motor behaviors, sleep, and eating. As there were only a few participants with a score > 0 for some of the 12 symptoms, participants were simply divided into those with and without symptoms of each domain, based on a total score of > 0 or 0. LC/SN contrast was compared between the two groups. For each participant, the scan closest to the date of the NPI was used for this analysis, and only those with a scan within a year were included.

### 2.7 Plasma sample assessment

Plasma samples were taken on the day of the MRI scan (*N* = 53), or within 1 year (*N* = 8). A cannula was first inserted, and then blood was drawn 10 min later with the patient supine, so as to allow for the autonomic response to needle insertion to subside. Participants were asked not to eat or drink at least 1 h before blood sampling and to abstain from caffeine and exercise on the day. Blood samples were collected using ethylenediaminetetraacetic acid‐coated tubes. After 30 to 120 min at room temperature, samples were centrifuged at 2500 g at 4°C for 20 min and frozen at −80°C. For a subgroup of 37 AD and 24 HC, plasma pTau217, NfL, and GFAP concentration was quantified using the ALZpath pTau217 and Simoa‐Neurology assays (Quanterix, Inc.).

### 2.8 Statistical analysis

All analysis was conducted using RStudio (Posit, PBC). Group differences in demographics were compared using *t*‐tests, Wilcoxon tests, and chi‐squared tests as appropriate. Linear mixed‐effects models using contrast measures from multiple timepoints were used to test for group and laterality differences in LC/SN contrast and to test the relationship of the two nuclei with age and with each other, additionally controlling for time from baseline scan, and with participant as a repeated measure term. Linear regression models were used to test the ability of a single LC/SN contrast measure per participant to predict contemporaneous ACE scores, plasma biomarkers, and JD (atrophy rate between the scan used to measure the LC contrast and a subsequent follow‐up scan). For comparing those with and without NPI symptoms for each domain, logistic regression was used. As a further exploratory analysis of the relationship between LC/SN contrast and contemporaneous ACE score, LC/SN values were converted to *z*‐scores relative to controls. *K*‐means clustering was then performed on these values and identified three clusters of patients, as recommended by the elbow method (Figure S3). For each outcome (ACE total and subdomain scores), a type III analysis of covariance (ANCOVA) was fitted with cluster as the primary predictor, plus nuisance co‐variates. Post hoc pairwise comparisons between clusters were obtained from estimated marginal means with Tukey adjustment within each outcome. For regression models, age, sex, and AD symptom duration (to account for disease stage), plus education for cognitive outcomes, were included as standard. We did not correct for multiple comparisons across the different ACE and NPI domains, plasma biomarkers, or side (left/right).

To estimate the annual rate of change in cognition over time, linear mixed‐effects models were employed to calculate individual participants’ rate of change. The equation used was

$$\mathrm{ADAS}-\mathrm{Cog}∼\mathrm{timefrombaseline}+\left(\mathrm{timefrombaseline}|\mathrm{participant}\right)$$

where “ADAS‐Cog” represents the score at a particular timepoint and “timefrombaseline” represents the time the score was taken relative to that participant's first ADAS‐Cog score. Fixed effects, indicating the average rate of change in ADAS‐Cog scores per year for the entire group, and the random effects, representing the individual variation in the rate of change per year, were extracted. The annual rate of change for each participant was then calculated by summing the fixed, group‐level rate of change and the individual's random slope. This approach enabled modeling of the group‐level trend in cognitive scores while considering the unique trajectory of each participant and their baseline cognitive function. The annual rate of change was then used as the dependent variable in a linear regression model with LC/SN contrast as predictor variable plus nuisance covariates. Additionally, a model was run including pTau217 for the 19 participants with these data available.

## 3 RESULTS

### 3.1 Participant characteristics

In total, 93 AD participants and 29 healthy older controls underwent MRI scanning (Table 1). AD had an ACE of 56.3/100 ± 17.6 (mean ± SD), which is considered “moderate dementia,”^54^ and had a symptoms duration of 5.2 ± 3.2 years (mean ± SD). Scores for the ACE subdomains in both groups are reported in Table 1. A subgroup of AD participants (*N* = 28) returned for a second scan after approximately a year, with 12 returning for a third scan a year later. On eight occasions (out of 162 scans), brainstem contrast imaging was not acquired as the participant did not tolerate the whole scan. Fourteen brainstem contrast scans could not be analyzed due to artifact, giving a total of 140 analyzable scans.

**TABLE 1:** Participant demographics.

	HC	AD	Statistic	*p*
Total included in analyses	29	93		
Age, years, mean (SD)	75.6 (6.1)	75.0 (8.2)	*t* = −0.41	0.68
Sex, percentage female	48.3	54.8	*χ* ^2^ = 0.52	0.47
ACE total, mean (SD)	*N* = 28, 95.8 (3.6)	*N* = 77, 56.3 (17.6)	*W* = −9.5	<0.001
Attention	17.5 (0.8)	10.1 (4.1)	*W* = −91.5	<0.001
Fluency	12.4 (1.4)	6.4 (3.4)	*W* = −93.5	<0.001
Language	25.6 (0.5)	19.1 (5.2)	*W* = −108	<0.001
Visuospatial	15.5 (0.8)	11.3 (4.1)	*W* = −248	<0.001
Memory	24.7 (1.8)	9.4 (5.2)	*W* = −26	<0.001
Full‐time education, years, mean (SD)	15.1 (3.9)	13.7 (3.0)	*W* = 857.5	0.13
Dementia symptoms, years, mean (SD)		5.2 (3.2)		

*Note*: Age and years of symptoms at first MRI visit for each participant. *T*‐test, Wilcoxon tests, and chi‐squared tests are used as appropriate. *p* values are uncorrected for multiple comparisons.

Abbreviations: Alzheimer's disease; ACE, Addenbrooke's Cognitive Examination; HC, healthy control; AD, SD, standard deviation.

### 3.2 Effects of AD and age on LC and SN integrity

**FIGURE 2:**
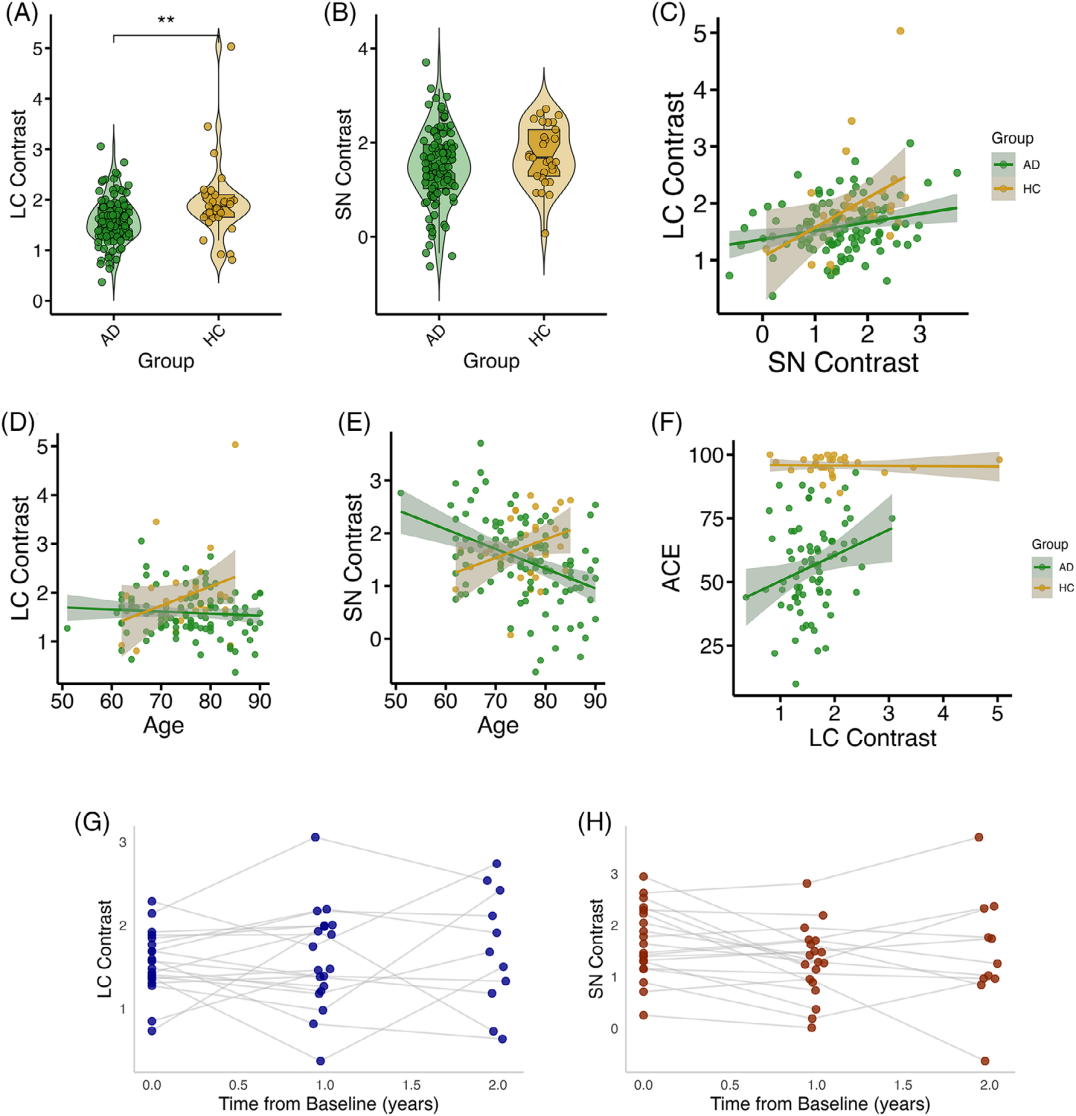
LC and SN integrity in AD and healthy aging. Violin plots showing LC (A) and SN (B) contrast by group. Data from all visits plotted, including multiple timepoints for 19 AD participants. Significance shown is the result of linear mixed‐effects models adjusting for covariates. **p < 0.01. (C) Scatter plot showing relationship between LC and SN contrast, split by group. (D and E) Scatter plot showing relationship between LC/SN contrast and age, split by group. (F) Scatter plot showing relationship between LC contrast and ACE, split by group. (G and H) Scatter plots showing LC/SN contrast over time, relative to baseline scan, in AD participants only. Values from same participant joined by gray line. There is no significant group level change over time for either measure. For (A–F), the shaded error bar shows standard error of the mean. For (C–F), the line of best fit through the raw data is plotted. AD, Alzheimer's disease (green); HC, healthy control (gold); LC, locus coeruleus; SN, substantia nigra.

Using mixed‐effects models, we compared the contrast in the LC and SN between the AD and control groups. To do this we utilized all analyzable scans (*N* = 140), including multiple visits from 19 AD participants. There was a significant reduction in LC contrast in AD (*b *= −0.39, SE = 0.12, *t*[125.0] = 3.26, *p* = 0.001) (Figure 2A). There was no significant effect of sex or age on LC contrast. For the SN there was no significant group difference (*b *= −0.25, SE = 0.15, *t*[121.7] = 1.59, *p* = 0.11) (Figure 2B). SN contrast decreased significantly with male sex (*b *= −0.30, SE = 0.13, *t*[103.8] = −2.21, *p* = 0.03) and age (*b *= −0.02, SE = 0.01, *t*[93.87] = −2.53, *p* = 0.01). There was a significant positive relationship between LC and SN contrast (*b *= 0.35, SE = 0.11, *t*[132.8] = 3.23, *p* = 0.002) but no group interaction (Figure 2C).

Contrast was significantly lower on the right side compared to the left for both the LC (*b *= −0.30, SE = 0.06, *t*[169.0] = −5.21, *p* < 0.001) and SN (*b *= −0.26, SE = 0.07, *t*[168.0] = −3.78, *p* < 0.001) (Figure S4). While neither nucleus showed a side‐by‐group interaction, the group difference between AD and HC was numerically larger for both nuclei for the left (LC: *b *= 0.46, SE = 0.14, *t*[119.6] = 3.27, *p* = 0.001; SN: *b *= 0.29, SE = 0.17, *t*[119.4] = 1.69, *p* = 0.09) than the right (LC: *b *= 0.32, SE = 0.14, *t*[130.1] = 2.32, *p* = 0.02; SN: *b *= 0.21, SE = 0.18, *t*[131.5] = 1.17, *p* = 0.24) (Figure S4).

As neuromelanin is known to increase with healthy aging^13^ but decrease in AD, we further explored the effect of aging of LC/SN contrast by looking at the interaction between age and group (Figure 2D,E). For the LC there was a significant interaction of age and group (*b *= 0.05, SE = 0.02, *t*[121.7] = 2.52, *p* = 0.01) driven by a non‐significant trend toward increasing LC contrast with age in HC (*b *= 0.04, SE = 0.03, *t*[26] = 1.590, *p* = 0.12) and decreasing in AD (*b *= −0.01, SE = 0.01, *t*[84.6] = −1.04, *p* = 0.30). For the SN, there was a stronger interaction (*b *= −0.03, SE = 0.01, *t*[89.6] = −3.47, *p* < 0.001) due to a borderline significant increase in contrast with age in HC (*b *= 0.04, SE = 0.02, *t*[26] = 1.93, *p* = 0.06) and a significant decrease in AD (*b *= −0.03, SE = 0.01, *t*[69.9] = −3.17, *p* = 0.002).

For the 19 AD participants with multiple neuromelanin MRI timepoints (eight with two, 11 with three), there was no significant effect of time from baseline scan on either the LC (*b *= 0.00, SE = 0.00, *t*[33.9] = 0.91, *p* = 0.37) or SN (*b *= 0.00, SE = 0.00, *t*[32.4] = −1.37, *p* = 0.18), indicating that at the group level, contrast values did not change over time (Figure 2G,H).

### 3.3 Relationship of LC and SN integrity to cognition

**FIGURE 3:**
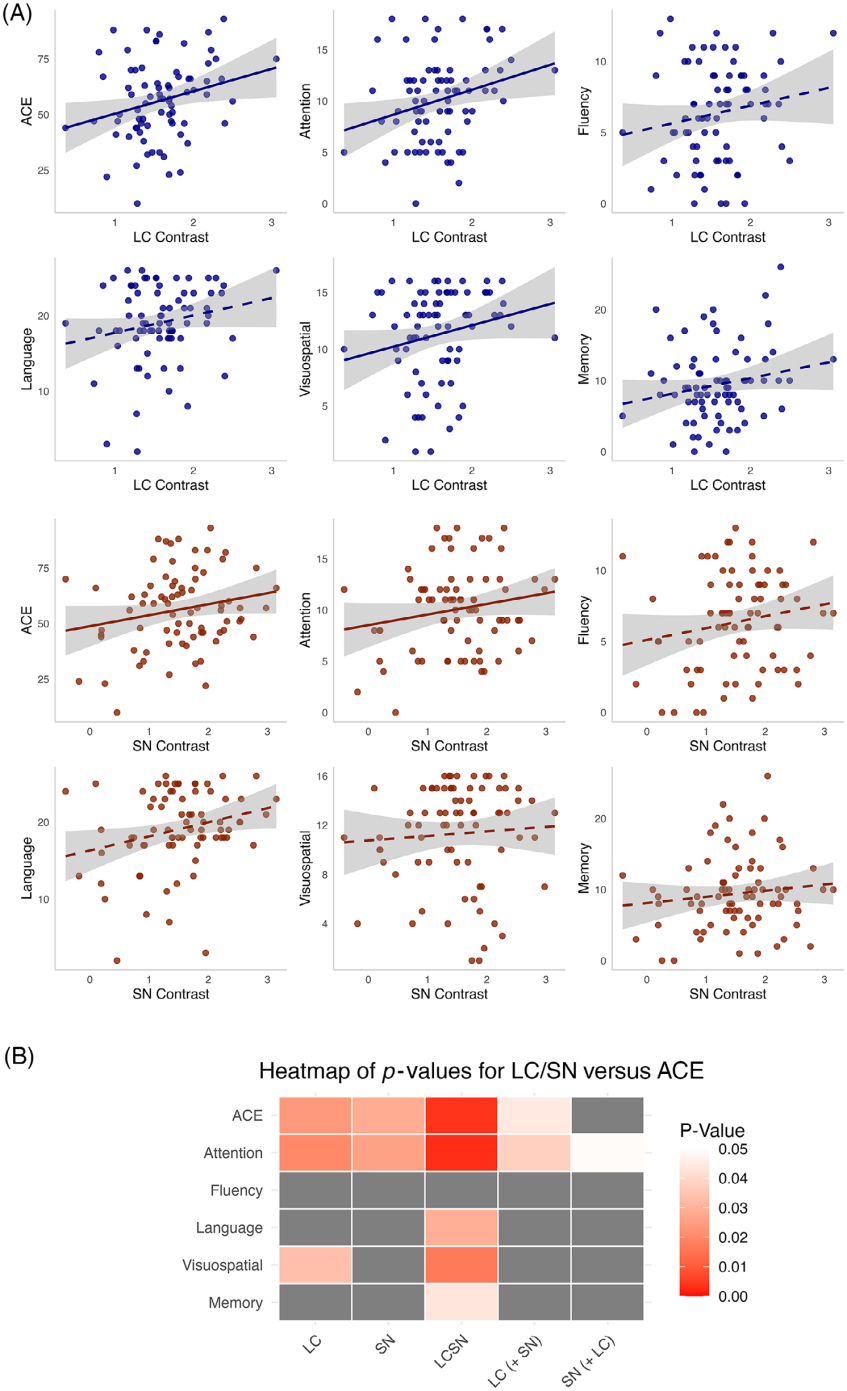
Relationship with ACE total score and subdomain scores. (A) Scatter plots showing relationship with ACE for LC (blue) and SN (brown) contrast in AD, one timepoint per participant. Significant relationships according to linear regression models run separately for each nucleus are shown with a solid line of best fit through the raw data. Shaded error bar shows standard error of the mean. (B) Heatmap showing the *p* values resulting from linear regression models. All models include age, sex, education, and length of symptoms as covariates. The LC (+ SN) column shows the *p* value related to the LC in the model in which both contrast values are included in the same model, and vice versa for SN (+ LC). LCSN is a single composite value derived from both nuclei. *p* > 0.05 marked in gray. ACE, Addenbrooke's Cognitive Examination total score; LC, locus coeruleus; SN, substantia nigra.

We examined the relationship between LC/SN integrity and cognition in all participants who had same‐day ACE and MRI data (76 AD, 28 HC). Linear regression revealed a significant interaction of group and LC integrity on total ACE (*b *= −10.41, SE = 5.08, *t*[96] = −2.05, *p* = 0.04). This was due to a significant positive relationship in AD (*b *= 9.71, SE = 4.20, *t*[70] = 2.31, *p* = 0.02), and no relationship in the HC (*b *= −0.22, SE = 0.91, *t*[23] = −0.25, *p* = 0.81) (Figure 2F). Given LC integrity and cognition were significantly related in the AD group only, we then ran a series of linear regression models to further explore the relationship with cognition in just that group (Figure 3, Table S1). For each model, the predictor was either LC or SN contrast, or LCSN composite contrast value, and the dependent variable was ACE total or subdomain score. LC showed a significant positive relationship with total ACE score (*b *= 9.71, SE = 4.2, *t*[70] = 2.31, *p* = 0.02), attention (*b *= 2.33, SE = 0.98, *t*[70] = 2.38, *p* = 0.02), and visuospatial ability (*b *= 2.16, SE = 0.99, *t*[70] = 2.17, *p* = 0.03). SN was significantly positively associated with total ACE score (*b *= 6.38, SE = 2.85, *t*[70] = 2.24, *p* = 0.03) and attention (*b *= 1.51, SE = 0.66, *t*[70] = 2.28, *p* = 0.03). The LCSN composite was significantly positively associated with scores in attention, language, visuospatial ability, and memory, but not fluency (Figure 3B) (see Table S1 for statistics).

As a sensitivity analysis, the preceding models were additionally run with total gray matter volume and estimated total intracranial volume as covariates. Gray matter volume was significantly associated with total ACE and all subdomain scores (except visuospatial ability), when accounting for LC and SN together or when accounting for the LCSN composite. The LC remained significantly associated with ACE score when accounting for SN and gray matter volume (*b *= 9.78, SE = 4.21, *t*[54] = 2.32, *p* = 0.02), while the associations with attention (*b *= 1.91, SE = 1.07, *t*[54] = 1.78, *p* = 0.08) and language (*b *= 2.45, SE = 1.29, *t*[54] = 1.9, *p* = 0.06) were trend level. The SN and LCSN composite were not associated with cognition when accounting for gray matter volume (see Table S1 for full statistics).

**FIGURE 4:**
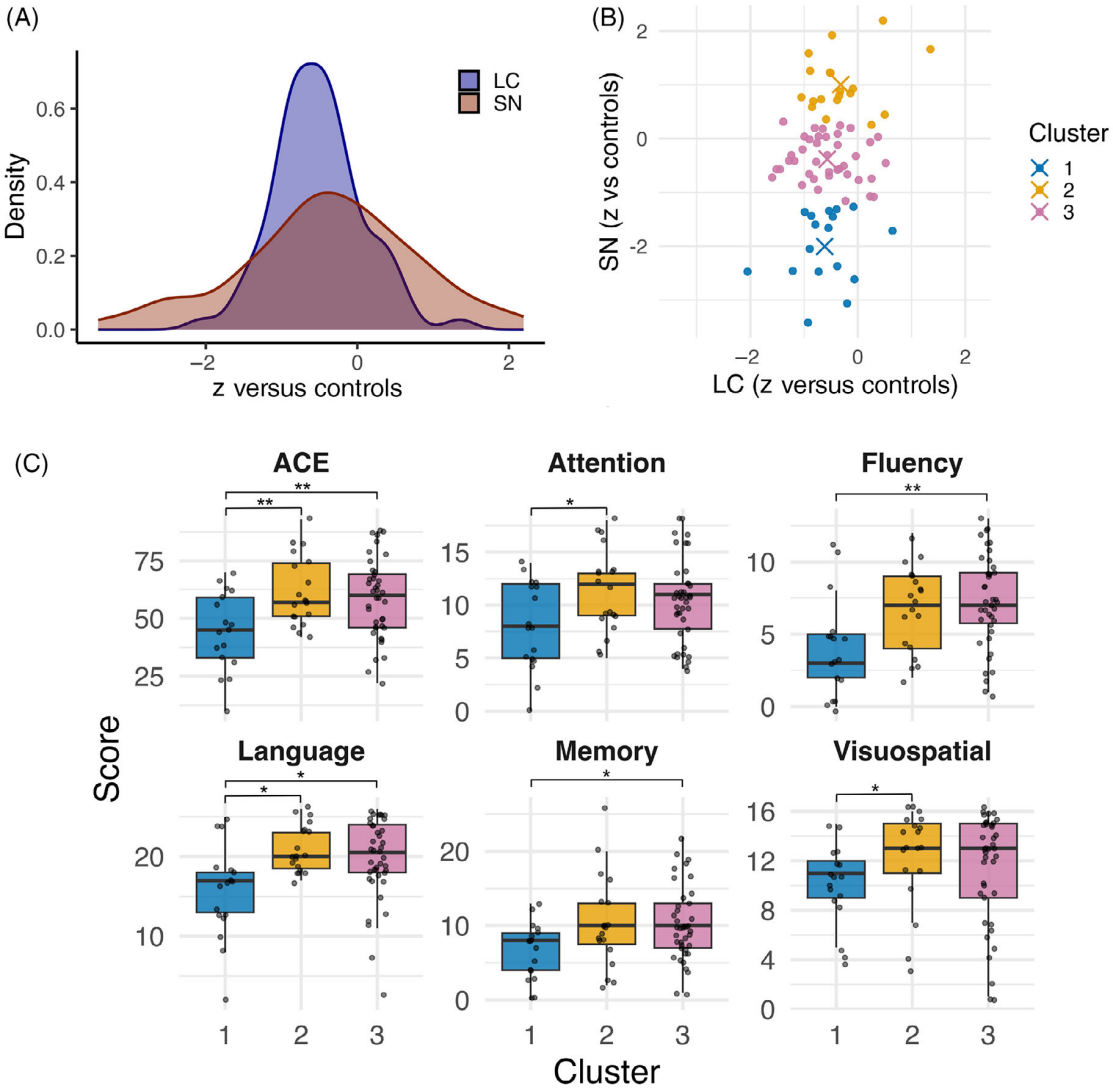
Clustering of AD participants and relationships with cognition (A) density plots showing distribution of LC and SN contrast values (converted to *z*‐scores relative to controls) in AD group. (B) Scatter plot showing clusters of AD participants based on their LC/SN contrast values. (C) Box plots showing ACE total and subdomain scores for each AD cluster. Significance shown is the result of analysis of covariance adjusting for covariates. Post hoc Tukey adjustment performed for comparisons between clusters within each ACE domain. *p < 0.05, **p < 0.01. ACE, Addenbrooke's Cognitive Examination total score; AD, Alzheimer's disease; LC, locus coeruleus; SN, substantia nigra.

In a further exploratory analysis, AD participants were grouped into three clusters using *k*‐means, based on their LC and SN values (“Methods”) (Figure 4A,B). Relative to controls, Cluster 1 showed a moderate reduction in LC contrast (−0.6 SD) with marked SN reduction (−2.0 SD); Cluster 2 had a milder LC reduction (−0.3 SD) and an increase in SN contrast (1.0 SD); Cluster 3 showed moderate LC reduction (−0.6 SD) with only mild SN reduction (−0.4 SD). The cognitive scores of each cluster were then compared. Cluster 1 had significantly lower total ACE scores compared to both Clusters 2 (*b *= −18.15, SE = 5.73, *t*[69] = −3.17, *p* < 0.01) and 3 (*b *= −14.34, SE = 4.6, *t*[69] = −3.12, *p* < 0.01). Cluster 1 also had significantly lower attention (*b *= −3.79, SE = 1.37, *t*[69] = −2.76, *p* = 0.02), language (*b *= −4.44, SE = 1.78, *t*[69] = −2.5, *p* = 0.04), and visuospatial ability (*b *= −3.45, SE = 1.41, *t*[69] = −2.46, *p* = 0.04) compared to Cluster 2; and significantly lower memory (*b *= −3.57, SE = 1.41, *t*[69] = −2.54, *p* = 0.04), fluency (*b *= −3.11, SE = 0.95, *t*[69] = −3.29, *p* < 0.01), and language (*b *= −3.63, SE = 1.43, *t*[69] = −2.54, *p* = 0.03) compared to Cluster 3 (Figure 4C). There were no differences between Clusters 2 and 3 (see Table S2 for full statistics).

### 3.4 Noradrenergic dysfunction and neuropsychiatric symptoms

**FIGURE 5:**
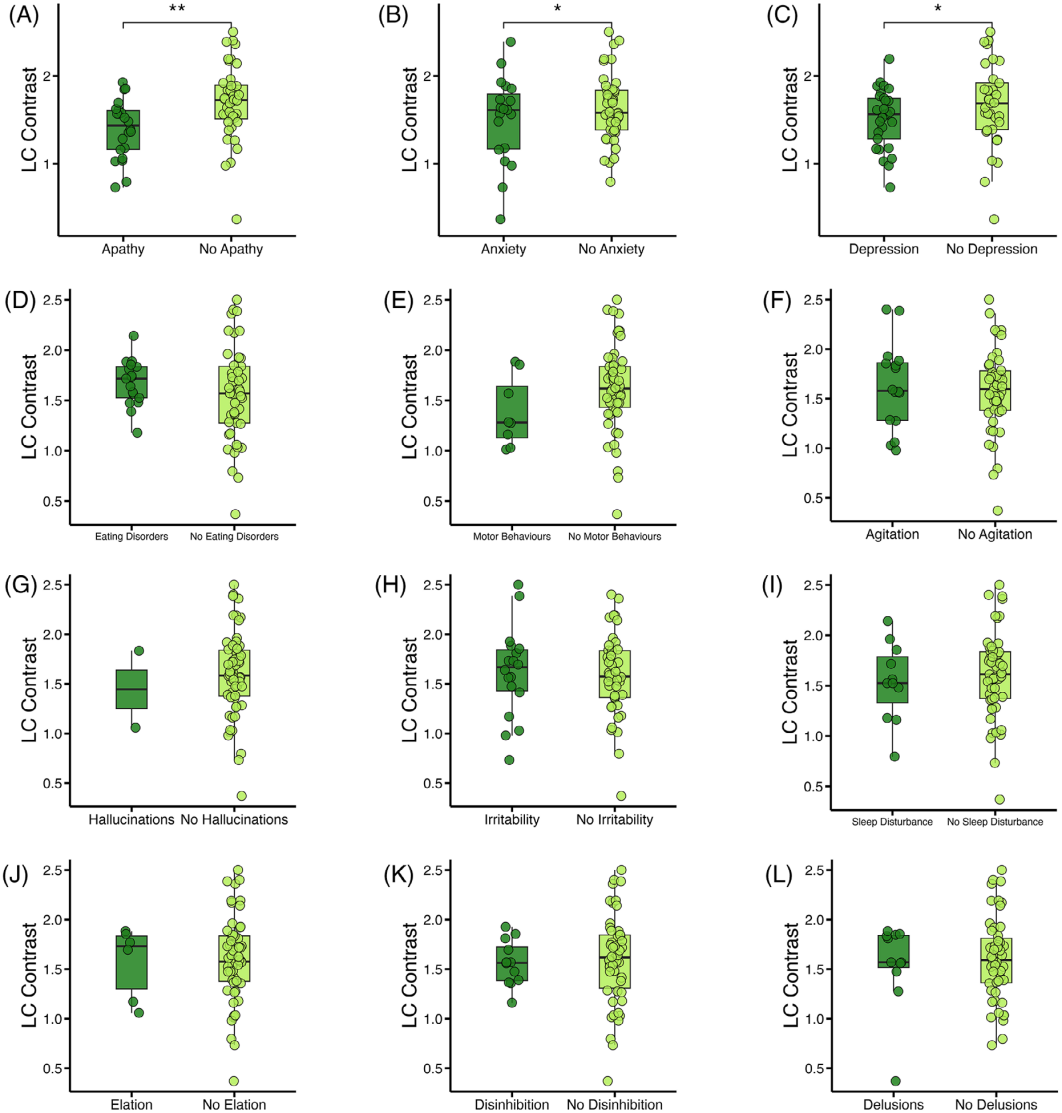
Using LC contrast to predict neuropsychiatric symptoms. Box plots showing significantly lower LC contrast for participants with versus without NPI score > 0 for (A) apathy, (B) anxiety, and (C) depression. (D–L) No difference seen in contrast between those with and without the other symptoms. Significance shown is the result of logistic regression adjusting for covariates. *p < 0.05, **p < 0.01. LC, locus coeruleus; NPI, Neuropsychiatric Inventory.

We investigated the relationship between LC and SN contrast and NPI scores from 62 AD participants. All NPI assessments were performed within 1 year of the scan with a gap in days of 95 ± 125 (mean ± SD). The participants with symptoms of apathy (*N* = 22/62, *b *= −2.24, SE = 0.85, *Z* = −2.63, odds ratio [OR] = 0.11, *p* < 0.01), anxiety (*N* = 19/62, *b *= −1.67, SE = 0.83, *Z* = −2.01, OR = 0.19, *p* = 0.04), and depression (*N* = 29/62, *b *= −1.58, SE = 0.74, *Z* = −2.13, OR = 0.21, *p* = 0.03), all had significantly lower LC contrast than those without (Figure 5) (uncorrected for multiple comparisons). All three significant symptom domains remained significant when including SN as a covariate, indicating an independent association of LC contrast. There was no difference in LC contrast between those with and without the other NPI symptoms. SN contrast was not associated with any neuropsychiatric symptoms (Table S3).

### 3.5 Catecholamines and Alzheimer's disease progression

#### 3.5.1 Plasma biomarkers

**FIGURE 6:**
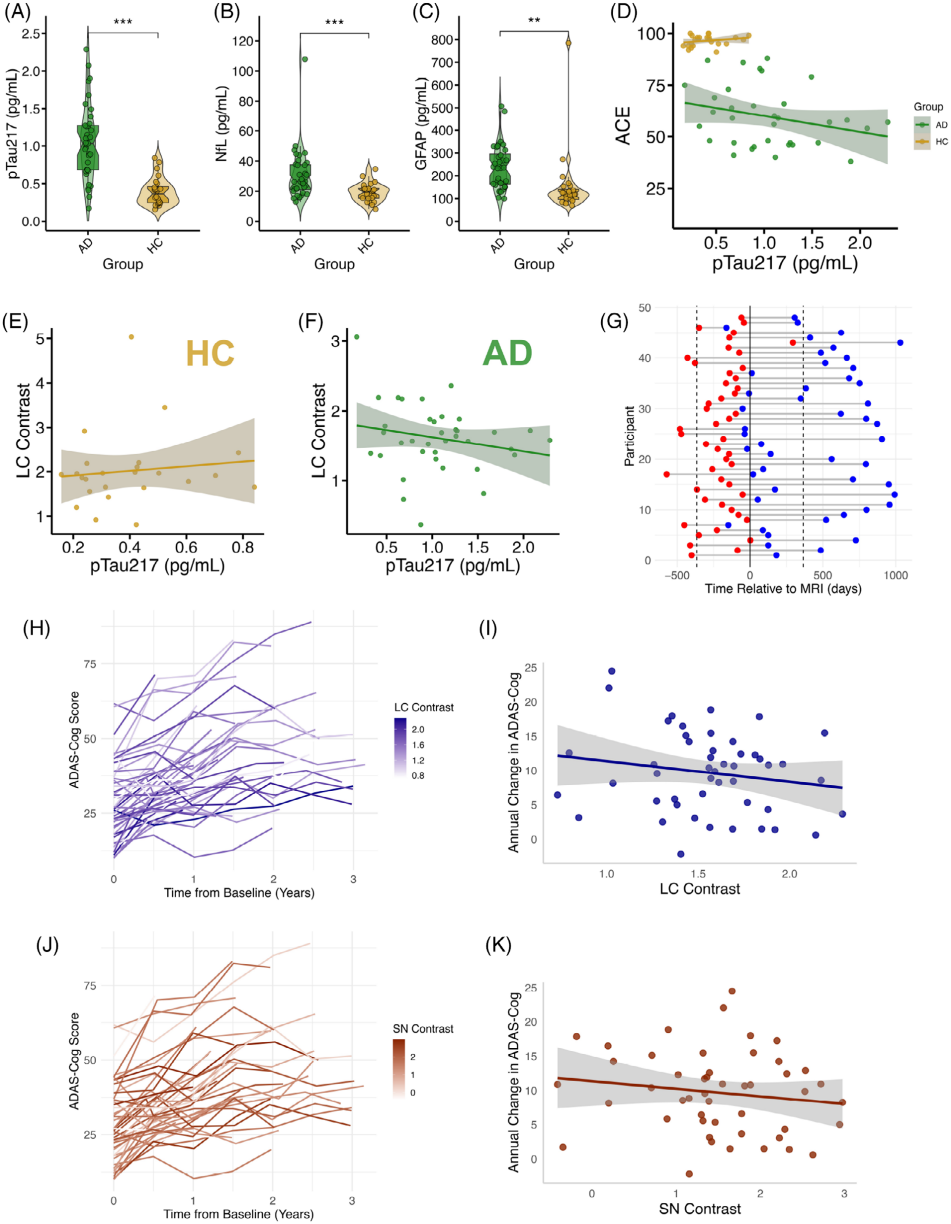
LC and SN integrity and disease progression. (A–C) Violin plots showing plasma pTau217, GFAP, and NfL by group. (D) Scatter plot showing relationship between plasma pTau217 and ACE, split by group. Shows a significant relationship across all participants, accounting for group, with no group interaction. (E and F) Scatter plot showing relationship between plasma pTau217 and LC contrast, split by group, showing significant relationship in AD but not HC. (D–F) Lines of best fit plotted through raw data. (G) Timeline of ADAS‐Cog relative to MRI scan used to compare LC contrast against rate of cognitive decline. The red and blue dots represent first and last ADAS‐Cog for each participant, respectively. Solid black vertical line represents the time of the MRI scan. Dashed black vertical line represents 1 year before/after the MRI scan. All scans fell during, or within 1 year of, the period of longitudinal cognitive testing. (H) Line graph representing each participant's ADAS‐Cog scores over time. Baseline taken as date of first ADAS‐Cog. Line color represents LC contrast value for that participant measured on a single MRI scan, with darker color indicating higher contrast. (I) Scatter plot showing LC contrast against annual rate of change in ADAS‐Cog score, with line of best fit through raw data. Panels (J) and (K) are as in (H) and (I) but for SN. Significant *p* values (< 0.05) in bold. All models include age, sex, and length of symptoms as covariates, plus education for cognitive decline. Shaded error bars show standard error of the mean. **p < 0.01, ***p < 0.001. ACE, Addenbrooke's Cognitive Examination; pTau217, phospho‐tau‐217; AD, Alzheimer's disease (green); ADAS–Cog, Alzheimer's Disease Assessment Scale–Cognitive; GFAP, glial fibrillary acidic protein; HC, Healthy control (gold); LC, locus coeruleus; NfL, neurofilament light; SN, substantia nigra.

Plasma biomarkers were quantified for 37 AD and 24 HC. One further AD participant's GFAP and NfL analysis failed. There was a significant difference between groups, accounting for age and sex, with elevated levels in AD for pTau217 (*b *= 0.64, SE = 0.11, *t*[57] = 6.03, *p* < 0.001), GFAP (*b *= 80.87, SE = 29.97, *t*[56] = 2.70, *p* < 0.01), and NfL (*b *= 13.04, SE = 3.09, *t*[56] = 4.21, *p* < 0.001) (Figure 6A–C).

There was a significant negative relationship between LC contrast and plasma pTau217 concentration, accounting for length of symptoms (*b *= −0.30, SE = 0.14, *t*[54] = −2.22, *p* = 0.03), with a significant group:LC contrast interaction (*b *= 0.34, SE = 0.17, *t*[54] = 0.17, *p* = 0.048). This was the result of a significant negative relationship in AD (*b *= −0.34, SE = 0.16, *t*[32] = −2.04, *p* = 0.05) but not HC (*p* > 0.05) (Figure 6E,F). There was a significant negative relationship between ACE and plasma pTau217 concentration (*b *= −8.02, SE = 3.78, *t*[50] = −2.12, *p* = 0.04), with no interaction of group (Figure 6D). There was no relationship between GFAP or NfL with LC contrast or ACE (all *p* > 0.05) (Table S4).

There was no relationship of pTau217, GFAP, or NfL with SN contrast (all *p* > 0.05) (Table S4). There was a significant SN:group interaction in predicting GFAP (*b *= −117.33, SE = 43.17, *t*[53] = −2.72, *p* < 0.01) due to a significant negative relationship in controls (*b *= −90.51, SE = 41.31, *t*[20] = −2.19, *p* = 0.04), but not in AD (*p* > 0.05).

#### 3.5.2 Cognitive decline rate

The ADAS‐Cog is typically used to show change in cognition in AD during clinical trials. It was conducted here at multiple timepoints, unrelated to the day of the MRI scanning, as a means of tracking rate of decline. The annual rate of change in ADAS‐Cog score was 9.69 ± 5.97 (mean ± SD; *N* = 48). LC contrast was significantly associated with cognitive decline rate with a lower contrast associated with a faster decline (*b *= −5.08, SE = 2.43, *t*[42] = −2.9, *p* = 0.04) (Figure 6H,I). When controlling for pTau217, the result also remained (*N* = 19, *b *= −7.16, SE = 3.05, *t*[12] = −2.35, *p* = 0.04), suggesting that the relationship between LC integrity and disease progression in clinical AD was independent of amyloid burden.

Lower SN contrast was also associated with faster cognitive decline rate (*b *= −2.4, SE = 1.06, *t*[42] = −2.27, *p* = 0.03) (Figure 6J,K); however this was not the case when accounting for pTau217 (N = 19, *b *= −0.18, SE = 1.73, *t*[12] = −0.11, *p* = 0.92). When including both SN and LC in the model, they both remained associated with rate of cognitive decline (LC: *b *= −4.74, SE = 2.33, *t*[41] = −2.04, *p* = 0.048; SN: *b *= −2.27, SE = 1.02, *t*[41] = −2.22, *p* = 0.03). The LCSN composite was a stronger predictor of cognitive decline than the individual nuclei (*b *= −4.57, SE = 1.56, *t*[42] = −2.93, *p* < 0.01).

#### 3.5.3 Atrophy rate

**FIGURE 7:**
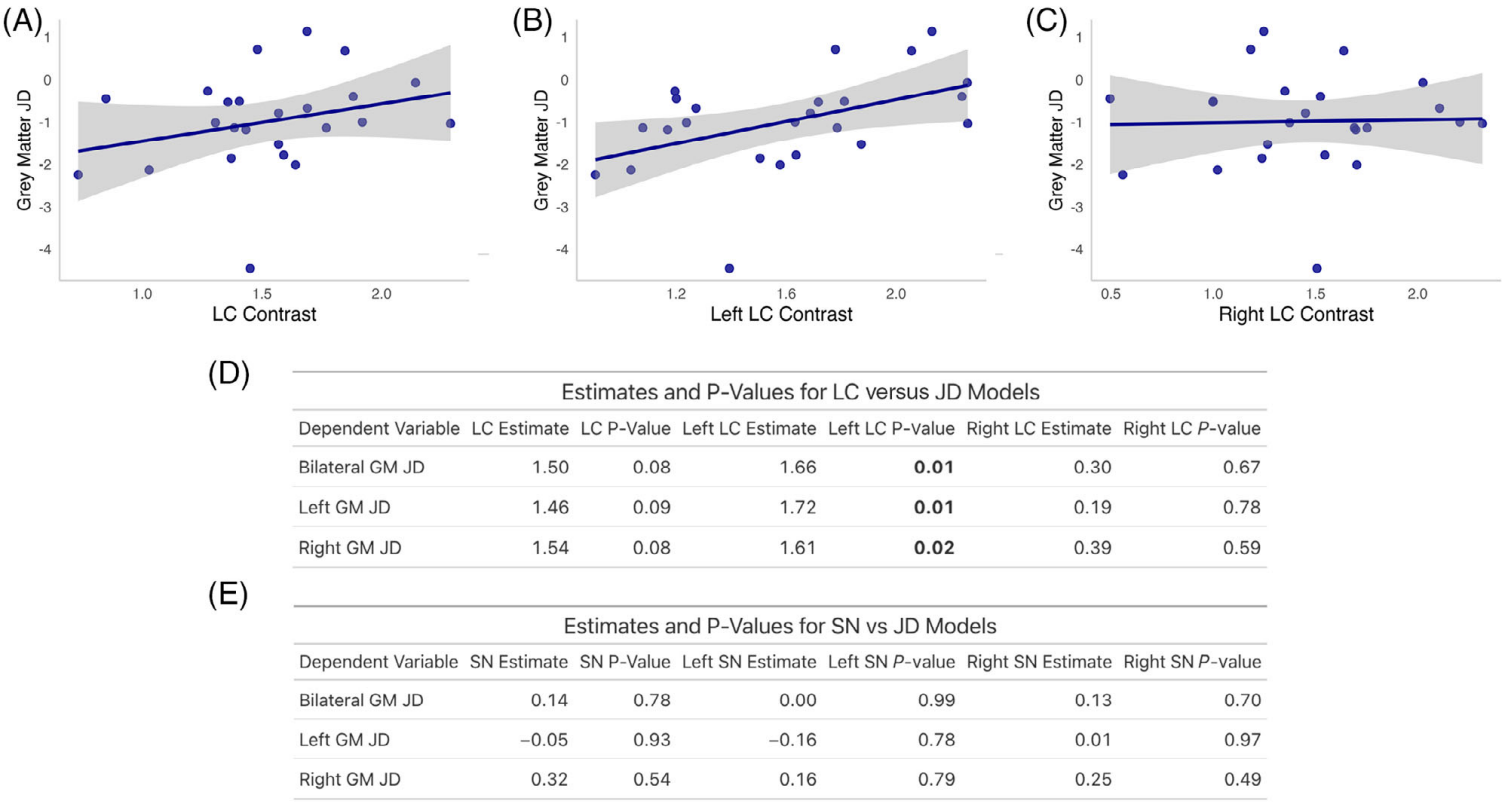
LC integrity and atrophy rate. (A–C) Scatter plots showing bilateral, left, and right LC contrast against bilateral GM JD, with line of best fit through raw data. (D) Table showing estimates and *p* values resulting from linear regression models between LC (above) and SN (below) contrast and cortical gray matter JD in AD participants. GM, gray matter; JD, Jacobian determinant; LC, locus coeruleus; SN, substantia nigra.

Having established a relationship between contrast in both nuclei and the rate of cognitive decline in AD, we looked at the relationship between nucleus contrast and subsequent atrophy rate (Figure 7A–C). This was done by calculating the JD for the bilateral cortical gray matter, and for the right and left side separately, in 28 AD participants, over 16.43 ± 6.96 months (mean ± SD). The bilateral gray matter JD was −0.93% ± 1.11 per year, and for the left and right it was −0.95% ± 1.10 and −0.91% ± 1.12 per year, respectively (mean ± SD). There was no difference between sides on *t*‐testing (*p* > 0.05). The relationship between LC contrast and bilateral gray matter (*b *= 1.50, SE = 0.80, *t*[19] = 1.87, *p* = 0.08), left gray matter (*b *= 1.46, SE = 0.83, *t*[19] = 1.77, *p* = 0.09), and right gray matter (*b *= 1.54, SE = 0.84, *t*[19] = 1.83, *p* = 0.08) JD showed a trend level relationship, that is, a trend to faster volume loss with lower contrast (Figure 7A,D).

In a further exploratory analysis, we also looked at the relationship between the contrast in each of the left and right LC and SN nuclei and the JD values. The left LC showed a significant relationship with all three JD measures (bilateral gray matter: *b *= 1.66, SE = 0.60, *t*[19] = 2.78, *p* = 0.01; left: *b *= 1.72, SE = 0.61, *t*[19] = 2.84, *p* = 0.01; right: *b *= 1.61, SE = 0.64, *t*[19] = 2.51, *p* = 0.02) (Figure 7B,D). The right LC showed no relationship with JD (Figure 7C,D). SN contrast, measured bilaterally or on either side, was not associated with gray matter JD (Figure 7E).

## 4 DISCUSSION

We found LC nucleus integrity, measured with neuromelanin MRI, was reduced in AD relative to HC, consistent with previous work.^15^, ^16^, ^17^, ^22^ In what we believe is the first application of this method to the SN in clinical AD, there was no group difference. While contrast in both nuclei related to global cognition, when accounting for both, only the LC still did so. LC integrity was lower in participants with apathy, anxiety, and depression symptoms, all of which are associated with noradrenergic function.^4^, ^55^ Notably, these analyses accounted for symptom duration, indicating a relationship to LC integrity per se rather than disease stage. LC integrity related to cognition even accounting for total gray matter volume.

MCI patients usually show deficits in a single cognitive domain, often memory, before the involvement of others.^56^ Accordingly, LC integrity has predominantly been associated with memory in prodromal/early stages.^19^, ^21^, ^57^ In this cohort however, most participants’ disease had progressed to a stage such that they displayed multidomain deficits. This may explain how associations of LC with attention and visuospatial ability were demonstrated here. In fact, both LC and SN integrity were related to attention when accounting for one another. Noradrenergic function is commonly associated with attention,^3^ and implications for AD have been described.^58^ Dopamine is also implicated in attentional control,^24^ specifically via prefrontal D_1_ receptors,^59^ and reduced SN contrast is associated with poorer attention and working memory in PD and healthy aging, respectively.^19^, ^60^ Interestingly, we found that a combined, equally weighted metric of both nuclei was the strongest predictor of global cognition. Also, a subgroup of AD participants with reduced SN integrity in addition to reduced LC integrity had worse cognition than those in which SN integrity was preserved. Together these findings point to the contribution of disruption to both systems to cognitive dysfunction in AD.

LC contrast was reduced in patients with symptoms of depression, anxiety, and apathy, when accounting for SN contrast. AD patients with depression have fewer LC neurons^61^ and a 10‐ to 20‐fold reduction in cortical noradrenaline.^62^ However, the mechanism underpinning this relationship is unclear and multifactorial.^25^ Specific neuropsychiatric symptoms are not present in all patients, and a recent meta‐analysis concluded that antidepressants are ineffective in dementia, likely due to the diversity of mechanisms.^55^ Better understanding of individual pathophysiology and targeting of subgroups likely to benefit from specific treatments is warranted.^55^

Apathy occurs in half of AD patients.^25^ Like depression, the cause is complex and likely secondary to both subcortical and cortical pathology, with both catecholaminergic systems implicated.^63^ Previous studies related LC MRI and dopaminergic SPECT imaging to apathy in PD and AD.^26^, ^64^, ^65^, ^66^ Symptom and mechanistic variability between individuals again highlight the need for biomarker‐led approaches. Unlike depression, meta‐analysis evidence shows a positive effect of noradrenergic drugs on apathy in AD,^67^ perhaps due to the dual dopaminergic‐noradrenergic action of some drugs.^63^

Previous work showed that pTau217 – a marker of amyloid and tau pathology^30^ – related to LC contrast in healthy older adults (not replicated here). Here, AD patients with lower LC integrity had higher pTau217. Because this holds when accounting for symptom duration, both biomarkers may reflect the variability in pathological burden between patients, rather than merely disease stage.^27^ This association was unique to pTau217; we did not see one with NfL or GFAP, both of which are sensitive but not specific to AD.^31^, ^32^

We also found a relationship between LC integrity and rate of cognitive decline and gray matter atrophy. AD is heterogenous, resulting in variable disease courses.^12^, ^68^ Multiple fluid and imaging biomarkers predict progression to AD from preclinical states, including LC contrast.^28^, ^69^, ^70^, ^71^ The relationship of LC integrity to rate of cognitive decline was independent of pTau217. This suggests it is not merely a corollary of cortical pathological burden but in fact drives disease progression independently. This was not true of the SN, for which the relationship between integrity and cognitive decline rate was not independent of pTau217.

Given extensive evidence for noradrenaline's neuroprotective effects, these results linking LC integrity to disease progression suggest a causal relationship. To summarize evidence reviewed elsewhere,^35^ noradrenergic loss induces proinflammatory cytokines (e.g., IL‐1β, TNF‐α, and IL‐6) – largely via β‐adrenergic receptors and cAMP pathways.^72^, ^73^ Also, noradrenaline reduces microglial Aβ clearance.^72^, ^73^ Interestingly, noradrenaline's neuroprotective effects are particularly prominent in the SN–dopaminergic system.^74^ Long‐term noradrenergic denervation reduces the number of SN dopaminergic neurons and affects behavioral function in rats,^75^ while noradrenaline depletion inhibits recovery of lesioned dopaminergic neurons.^76^ Therefore, loss of the neuroprotective effects of noradrenaline may lead to dopaminergic dysfunction, exacerbating symptoms.

This evidence evokes the possibility that therapeutically increasing noradrenergic tone may have neuroprotective, even disease‐modifying, effects. In a recent trial testing the potential AD‐modifying effects of the noradrenaline reuptake inhibitor atomoxetine, CSF total tau and pTau181 were reduced, while temporal lobe metabolism increased versus placebo.^77^ There was no effect on cognition or atrophy, perhaps as trial duration was only 6 months. Alongside anti‐amyloid therapies, drugs promoting tau removal are being trialed.^78^ If effective in slowing or even reversing LC damage and restoring noradrenergic function, these therapies may generate a neuroprotective positive feedback loop.

The LC integrity–cognition relationship may actually be bidirectional. It is believed that mental stimulation is protective against later‐life decline. In 2013, Robertson proposed that noradrenaline is key in mediating this cognitive reserve.^79^ Lifestyle factors that contribute to cognitive reserve stimulate noradrenergic activity, which Robertson claimed provides benefit through compensation and/or modification. In healthy people, established proxies of reserve relate to LC contrast.^80^ Also, LC tangle density is associated with lower social activity independent of amyloid and tau burden, and its relationship with cognition is partially mediated by reduced social activity.^81^ Cognitive reserve may not only protect against developing AD but also influence the disease course. LC integrity, as a biological marker of cognitive reserve, may protect against progression, even in symptomatic AD.

It is unclear why left LC was more strongly associated with atrophy. Although there are functional differences between left and right LC,^82^ projections from both are contra‐ and ipsilateral,^83^ and left LC does not accrue more tau or neuronal loss in AD.^84^ We note that Siemens scanners (used here) systematically produce higher left‐sided contrast.^13^ It is therefore possible that the stronger results relating to the left here (and elsewhere^23^) are due to higher contrast providing a better signal‐to‐noise ratio, as evidenced by the larger group difference seen on the left.

Unlike the LC–noradrenergic system, the extent of group‐level SN changes in AD remains unclear. Our null result may reflect insufficient statistical power, particularly if only a subgroup is affected.^8^, ^9^ This is reflected in the clustering analysis in which three subgroups of patients were identified. Each subgroup had similarly reduced LC integrity, but mildly increased, mildly decreased, or markedly decreased SN integrity, relative to controls. As a consequence, the spread of SN values relative to controls was considerably wider than for the LC (Figure 4A), perhaps reflecting the inconsistent presence of pathology in the SN, relative to the LC. Additionally, SN‐dopaminergic pathology in AD may not result in sufficient SN cell death to reduce neuromelanin contrast.^14^ Recent neuropathology work identified multiple mechanisms underlying the selective vulnerability of LC versus SN neurons, especially in early disease.^85^ In particular, LC's elevated cholesterol demand may cause inadvertent uptake of toxic Aβ. Nevertheless, while in health SN neuromelanin accumulates with age, we showed a negative relationship with age in AD. This interaction of age and disease suggests accelerated cell death in AD (Figure 2E).

Compared to cognitive decline, the lack of predictive value of the SN for subsequent atrophy rate highlights that the association between atrophy and cognition is not absolute. Axonal injury and pathological burden – leading to network disruption and synaptic dysfunction – represent alternative factors that may show a closer temporal relationship to cognitive changes than atrophy.^86^ Cognitive reserve also explains the non‐linear association between neurodegeneration and symptoms to some degree. It may also be that dopamine does not have the same neuroprotective effect and is therefore not a factor in the rate of neuronal loss.

Our work had the following methodological limitations. The methods of extracting contrast ratio values for the LC and SN were different. We used a semi‐automated method for the SN to avoid rater bias, in line with prior work.^87^, ^88^, ^89^, ^90^ For the LC, we used manual segmentation because its much smaller size,^91^, ^92^ especially in neurodegenerative populations, makes automated alignment less reliable than the SN.^93^ This is consistent with most clinical AD studies.^15^, ^16^, ^22^, ^94^ Importantly, however, it should be noted that the imaging sequence and equation used to calculate contrast ratio for both nuclei were identical. Second, to fully elucidate the specific contribution of both neuromodulatory systems to cognitive subdomains, more detailed cognitive assessment tools could be used.^95^ Third, our follow‐up period may have been too short to detect individual changes in LC contrast, previously observed over 2.5 years.^96^ Fourth, regarding analysis of nuclei integrity in relation to rate of cognitive decline, the scans were not aligned to the first cognitive test. This single, cross‐sectional measure was related to concurrent rate of decline but cannot be said to have definitively shown prospective predictive utility.

In conclusion, we conducted a multifaceted study of LC and SN integrity in relation to cognitive and neuropsychiatric symptoms in AD. Extending previous literature, we examined catecholaminergic function across a range of disease severity. LC integrity was reduced at the group level. Overall, there was no group difference in SN integrity, although both nuclei may be affected in a subgroup of AD. Both nuclei were related to attentional performance when accounting for one another. Further, LC integrity related to pTau217, confirming its relevance throughout the disease course. LC integrity not only reflects broader pathological burden but also predicts cognitive decline and, using the left side, the rate of gray matter atrophy. Given known neuroprotective effects of noradrenaline, our results suggest that LC dysfunction – more so than, and when controlling for, SN – accelerates the disease. Therefore, therapeutic modulation and/or protection of the noradrenergic system may be disease modifying.^77^

## CONFLICT OF INTEREST STATEMENT

Paresh A. Malhotra is lead for a NIHR‐funded trial with drug/placebo provided by Takeda Pharmaceuticals and sits on the Data Monitoring Committee for a trial carried out by Johnson & Johnson. He is vice chair of the Alzheimer's Society Research Strategy Council and NIHR Specialty Lead for Dementia and Neurodegeneration, Research Delivery Network. He is also an independent member of a data monitoring committee. All other authors have nothing to disclose. Author disclosures are available in the Supporting Information.

## Supporting information



Supporting Information

Supporting Information

## Data Availability

The Minder and PCNorAD studies were approved by the London‐Surrey Borders Research Ethics Committee (19/LO/0102) and London‐Central Research Ethics Committee (18/LO/0249), respectively. All participants with the capacity to consent provided written informed consent for participation and for their data to be included. Those without capacity were enrolled in accordance with the Mental Capacity Act (2005) on recommendation of an assigned consultee. Data are available from the corresponding author upon reasonable request.

## 1

** Acknowledgement list for collaborators from the UK Dementia Research Institute (UK DRI) Care Research & Technology (CR&T) Centre publications using the MINDER core data set**

**Leadership and Management Infrastructure**:

Centre Director: Professor David Sharp

Centre and Research Commercialisation Manager: Danielle Wilson

Health and Social Care Lead: Sarah Daniels

Clinical Lead: Professor Ramin Nilforooshan

General Practice Lead: Dr. David Wingfield

Human‐Centred Design Lead: Matthew Harrison

Movement Data and Living Lab Lead: Dr. Shlomi Haar

Data Science Lead: Nora Joby

Scientific Project Manager: Dr. Mara Golemme

Scientific Project Manager: Dr. Stephanie Lietz

Scientific Project Manager Dr. Margherita Tecilla

**Group Leaders**: Professor David Sharp, Professor Payam Barnaghi, Professor Paul Freemont, Professor Ravi Vaidyanathan, Professor Tim Constandinou, Dr. Gregory Scott, Imperial College London

Professor Derk‐Jan Dijk, University of Surrey, Guildford, United Kingdom.

**Emerging Leaders**: Dr. Shlomi Haar and Dr. Pete Lally

**Associate Members**: Professor Paresh Malhotra, Dame Professor Louise Robinson and Professor Adam Hampshire

**Groups: Behaviour and Cognition led by Prof David Sharp**

Affiliation: Imperial College London, Brain Sciences

Michael David

Martina Del Giovane

Neil Graham

Magdalena Kolanko

Helen Lai

Lucia M Li

Mark Crook Rumsey

Emma Jane Mallas

Alina‐Irina Serban

Eyal Soreq

Abidemi Otaiku

Megan ParkinsonThomas Parker

Success Fabusoro

Emily Beal

Julian Jeyasingh Jacob

Gaia Frigerio

Anastasia Mirza‐Davies

Ethan de Villiers

**Bioelectronic Systems led by Professor Timothy Constandinou**

Affiliation: Imperial College London, Electronic Engineering Alan Bannon

Danilo Mandic

Ziwei Chen

Charalambos Hadjipanayi

Ghena Hammour

Bryan Hsieh

Amir Nassibi

Adrien Rapeaux

Ian Williams

Maowen Yin

Niro Yogendran

**Robotics and AI interfaces led by Professor Ravi Vaidyanathan**

Affiliation: Imperial College London, Mechanical Engineering

Maria Lima

Alina‐Irina Serban

Ting Su

Melanie Jouaiti

Maitreyee Wairagkar

Carlos Sebastian Castillo

Panipat Wattansiri

Thomas Martineau

Mayue Shi

Tianbo Xu

Alejandro Valdunciel

Reneira Seeamber

Annika Guez

Zehao Liu

Saksham Dhawan

**Machine intelligence led by Professor Payam Barnaghi**

Affiliation: Imperial College London, Brain Sciences

Nan Fletcher‐Lloyd

Samaneh Kouchaki

Alexander Capstick

Chloe Walsh

Louise Rigny

Ruxandra Mihai

Marirena Bafaloukou

Jin Cui

Ann‐Kathrin Schalkamp

Yu Chen

Tianyu Cui

Nivedita Bijlani

**Point of care Diagnostics led by Professor Paul Freemont**

Affiliation: Imperial College London, Infectious Disease

Michael Crone

Kirsten Jensen

Martin Tran

Thomas Adam

Raphaella Jackson

Alexander Webb

David Wingfield

**Sleep and Circadian led by Professor Derk Jan Dijk**

Affiliation: University of Surrey, The Surrey Sleep Research Centre as part of the School of Biosciences

Anne C Skeldon

Kevin Wells

Ullrich Bartsch

Ciro Della Monica

Kiran K G Ravindran

Victoria L Revell

Hana Hassanin

James Woolley

Iris Wood‐Campar

Sarmad Al Gawwam

Aravind Kumar Kamaraj

Marta Pineda Messina

**Brain and movement led by Dr. Shlomi Haar**

Affiliation: Imperial College London, Brain SciencesNathan Steadman

Federico Nardi

Cosima Graef

Alena Kutuzova

Assaf Touboul

Nicolas Calvo Peiro

Jenna Yun

Sean Carr

Uri Rosenblum‐Belzer

**Groups: Computational Neurology led by Dr. Gregory Scott**

Affiliation: Imperial College London, Brain Sciences

Adela Desowska

Anastasia Gailly de Taurines

Ruxandra Mihai

Nina Moutonnet

**Helix Centre: Human Centred Design led by Matthew Harrison**

Affiliation: Imperial College London, Helix Centre and The Royal College of Art

Matthew Harrison

Sophie Horrocks

Victoria Simpson

Brian Quan

**Software engineering team**

Affiliation: Imperial College London, Brain Sciences

Mark Woodbridge

Anna Joffe

Ethan de Villiers

Amer Marzuki

Ramsheed Abdul Rahim

**Site Investigators and Key Personnel**:

**Name and Affiliation: Surrey and Borders Partnership NHS Foundation Trust (Site and Sponsor)**

Chief Investigator: Professor Ramin Nilforooshan

Research and Development Managers: Jessica True, Olga Balazikova

Research Co‐ordinator: Chloe Walsh

Clinical Monitoring Team: Nicole Whitethread, Matthew Purnell, Vaiva Zarombaite, Lucy Copps, Olivia Knight, Gaganpreet Bangar, Sumit Dey, Chelsea Mukonda, Jessica Hine, Luke Mallon, Saijal Jhala, Oliver Sargentoni, Amy Alves, Mahan Heydari

**Name and Affiliation: Hammersmith and Fulham Partnership**

Principal Investigator: Dr. David Wingfield

Research Nurse / Paramedic: Monica Morim

Clinical Studies Officers/Research Technicians: Anesha Patel, Ruby Lyall, Sanara Raza, Success Fabusoro, Gaia Frigerio, Maria Rasulo, Catalina Chavarro Novoa, Martynas Stonkus, Prital Patel, Zara Prem

Research Allied Healthcare Professionals: Naomi Hassim, Pippa Kirby

London Borough of Hammersmith and Fulham Support: Assistive Technology: John Patterson Business Development: Mike Law

Social Services OT: Andy Kenny

**Name: Minder Digitally Enabled Care for Dementia (MinderCare)**

Affiliation: Imperial College Healthcare NHS Trust, Imperial College London

Principal Investigator: Professor David Sharp

Co‐Investigators (not listed elsewhere) James Bird, Sarah Pearse

MinderCare Clinical Team: Joanna James, Janibo Amade Cassimo, Aglaja Dar, Pandora Wright, Lucia Li, Anastasia Mirza‐Davies, Julian Jeyasingh Jacobs, Zinca Zecevic, Sarah Daniels, David Wingfield

Research Technicians: Success Fabusoro, Gaia Frigerio, Maria Rasulo, Catalina Chavarro Novoa, Emma Burroughs
