# Supplementary material for: Catecholaminergic nucleus integrity and Alzheimer's pathology, symptoms, and progression
Source: Alzheimers Dement. 2025 Sep 29;21(10):e70749. doi: 10.1002/alz.70749 (PMC12479218; doi:10.1002/alz.70749)
Supplement: Supplementary file 1 — Supporting Information [file ALZ-21-e70749-s002.docx]

**Supplementary Materials**

**Locus coeruleus contrast ratio quantification**

**A)**

**B)**


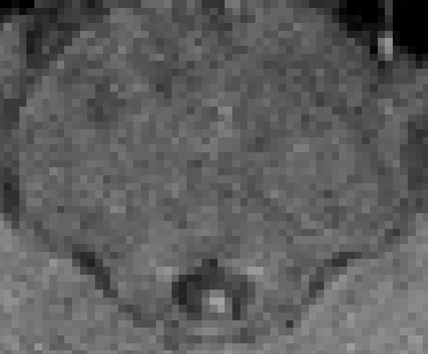

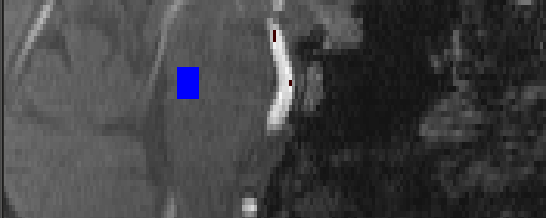

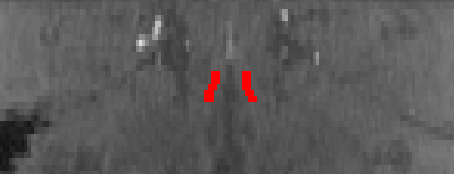

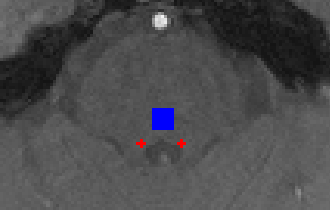


***Supplementary Figure 1. Method of sampling contrast in the locus coeruleus.*** ***A)*** *Example of location of locus coeruleus (red) and reference region (blue) in the axial (left), coronal (top right), and sagittal (bottom right) planes.* ***B)*** *Magnified axial slice highlighting 5 voxel locus coeruleus regions (red) for the left and right centred at the brightest voxel, and 7x7 voxel reference region (blue) nearby in the dorsal pons.*

**Substantia nigra contrast ratio quantification**


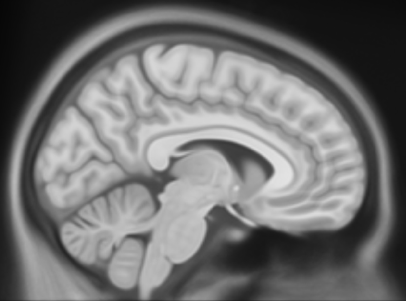

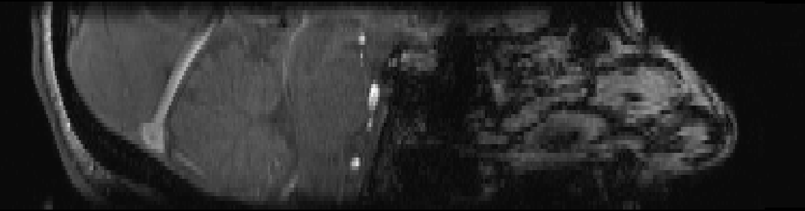

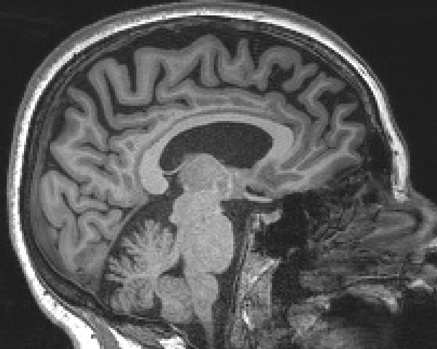

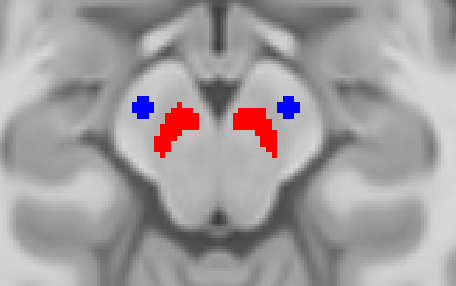

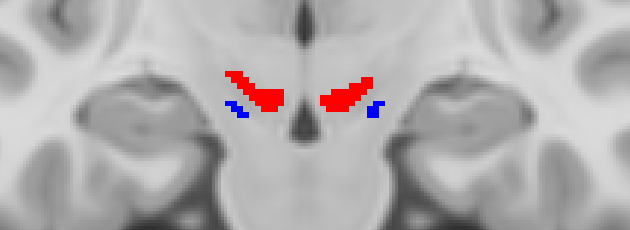


1

2

3


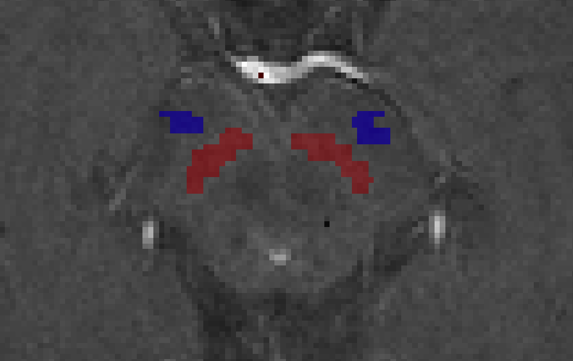


**A)**

**B)**

**C)**

***Supplementary Figure 2. Method of sampling contrast in the substantia nigra.*** ***A)*** *MNI template (Step 1) and subject brainstem scan (Step 2) warped to subject T1 (sagittal slices shown from a representative subject).* ***B)*** *Probabilistic MNI-space substantia nigra mask^46^ (red) and reference region (blue) masks shown on coronal (left) and axial (right) slices of the MNI template. For (Step 3), transform from (Step 1) and inverse transform from (Step 2) applied to masks.* ***C)*** *Example axial slice from brainstem scan of the same subject with masks overlayed. Masks checked to ensure appropriate alignment in subject space and then contrast sampled from within the masked regions.*


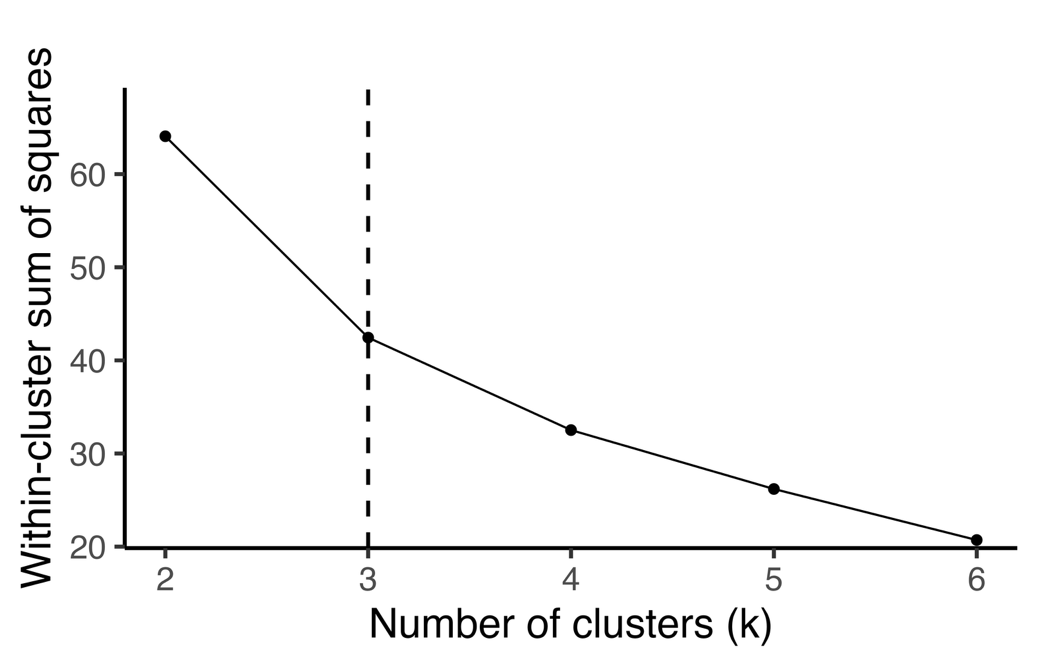


***Supplementary Figure 3.* K*-means clustering of AD participants based on LC and SN contrast.*** *Elbow plot showing how the sum of squared distances decreases with increasing number of clusters (*k*). The steep drop from* k*=2 to* k*=3 suggests that adding more clusters beyond that point yields diminishing returns in terms of within-cluster variance reduction. AD=Alzheimer’s disease; LC=locus coeruleus; SN=substantia nigra.*


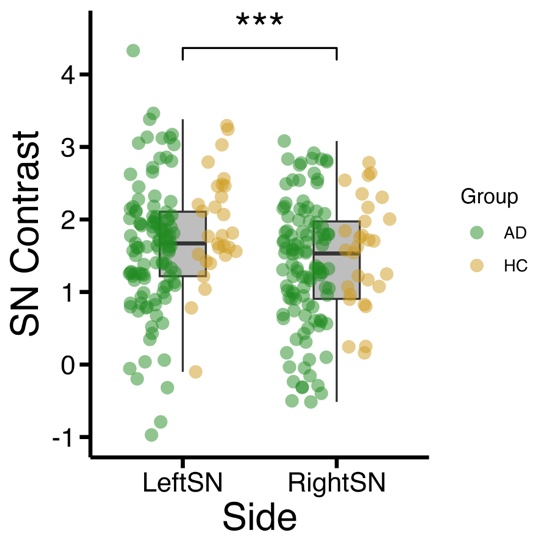

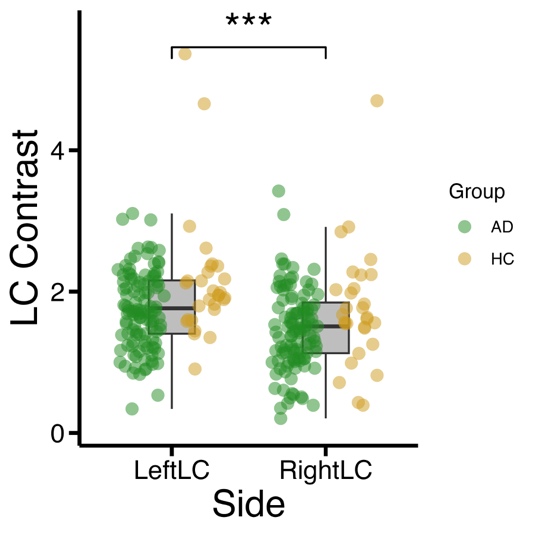


**B)**

**A)**

***Supplementary Figure 4.*** *Boxplots comparing the contrast in the left and right LC* ***(A)*** *and SN* ***(B)****, split by group. Significance shown is the result of linear mixed-effects models adjusting for covariates. ***P<0.001. There was no group by side interaction for either nucleus. HC=Healthy control (gold); AD=Alzheimer’s disease (green); LC=locus coeruleus; SN=substantia nigra.*

***
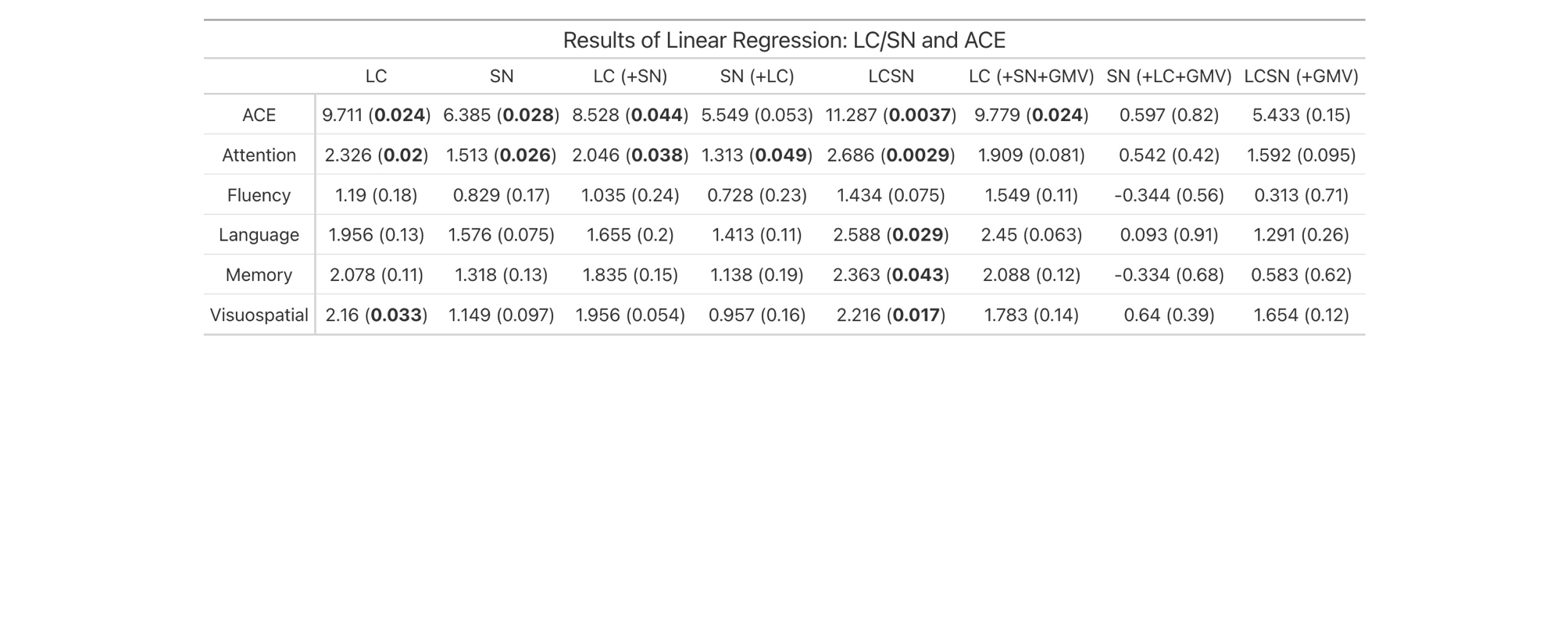
***

***Supplementary Table 1.*** *Table showing the estimates (P-values) resulting from linear regression models between ACE total as well as sub-domain scores, and LC/SN contrast, in Alzheimer’s disease participants. All models include age, sex, education, and length of symptoms as covariates. The LC (+ SN) column shows the P-value related to the LC in the model in which both contrast values are included in the same model, and vice versa for SN (+LC). Columns with (+GMV) also account for grey matter volume and estimated total intracranial volume. LCSN is a single composite value derived from both nuclei. Significant P-values (<0.05) in bold. ACE=Addenbrooke’s Cognitive Examination; LC=locus coeruleus; SN=substantia nigra.*


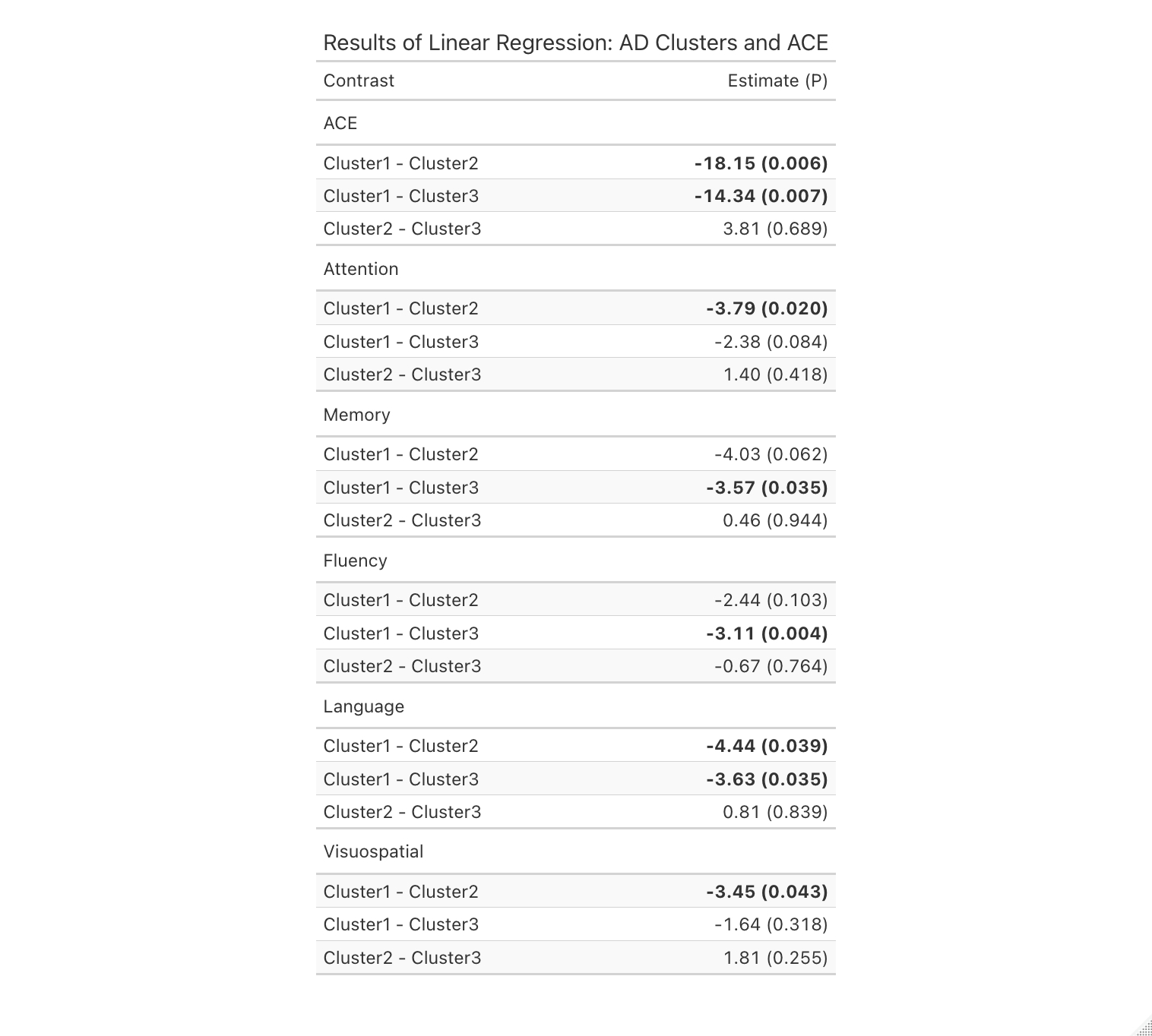


***Supplementary Table 2.*** *Table showing the estimates (P-values) resulting from linear regression models between ACE total as well as sub-domain scores, and LC/SN clusters of Alzheimer’s disease participants. All models include age, sex, education, and length of symptoms as covariates. Significant P-values (<0.05) in bold. ACE=Addenbrooke’s Cognitive Examination; LC=locus coeruleus; SN=substantia nigra.*


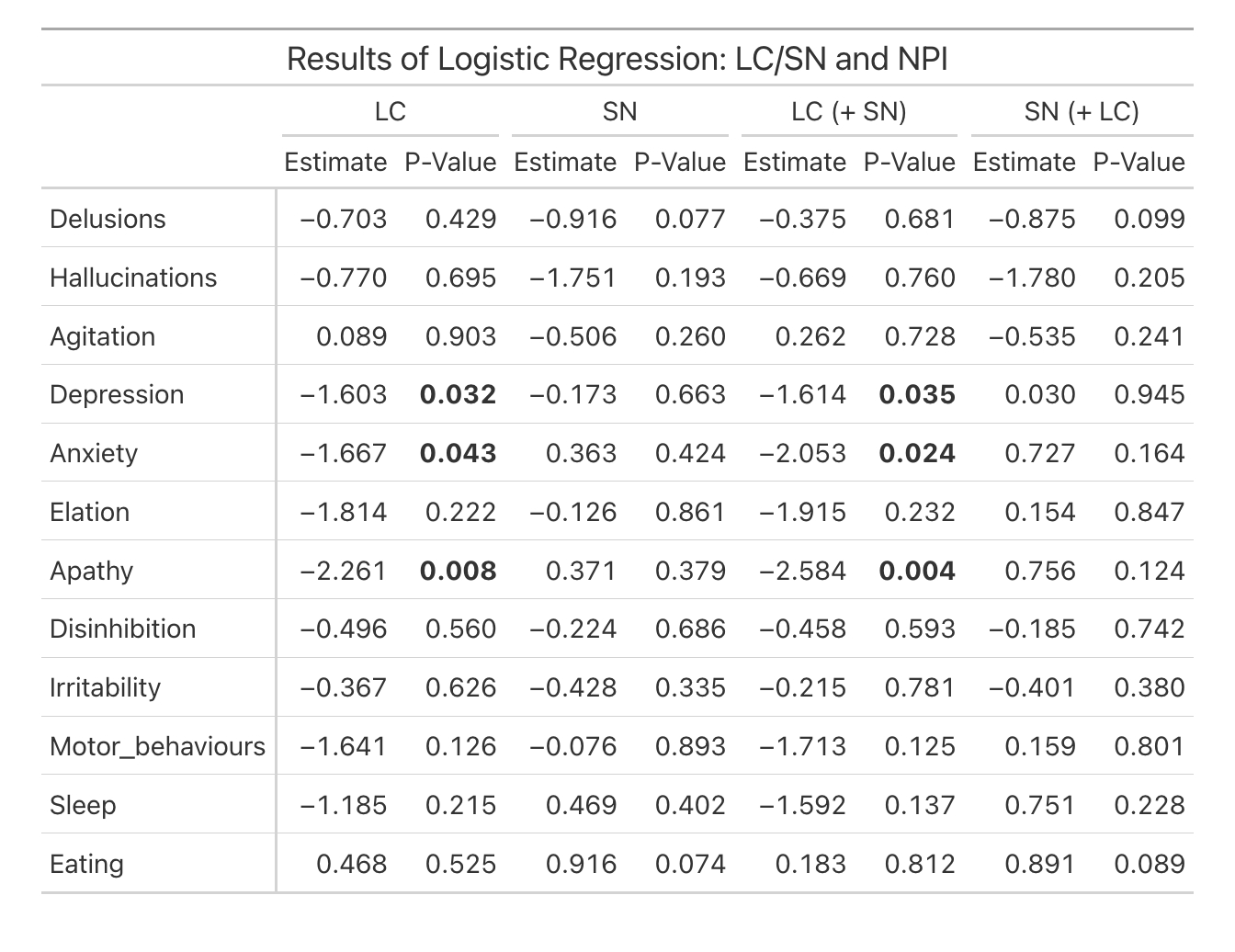


***Supplementary Table 3.*** *Table showing the results from logistic regression models comparing LC and SN contrast in participants with versus without a sub-domain score >0 on the neuropsychiatric inventory. Each model run accounting for age, sex, and length of symptoms. Additional analysis including the other nucleus (LC or SN) as a covariate also run. Significant P-values (<0.05) in bold. NPI=Neuropsychiatric Inventory; LC=locus coeruleus; SN=substantia nigra.*

*
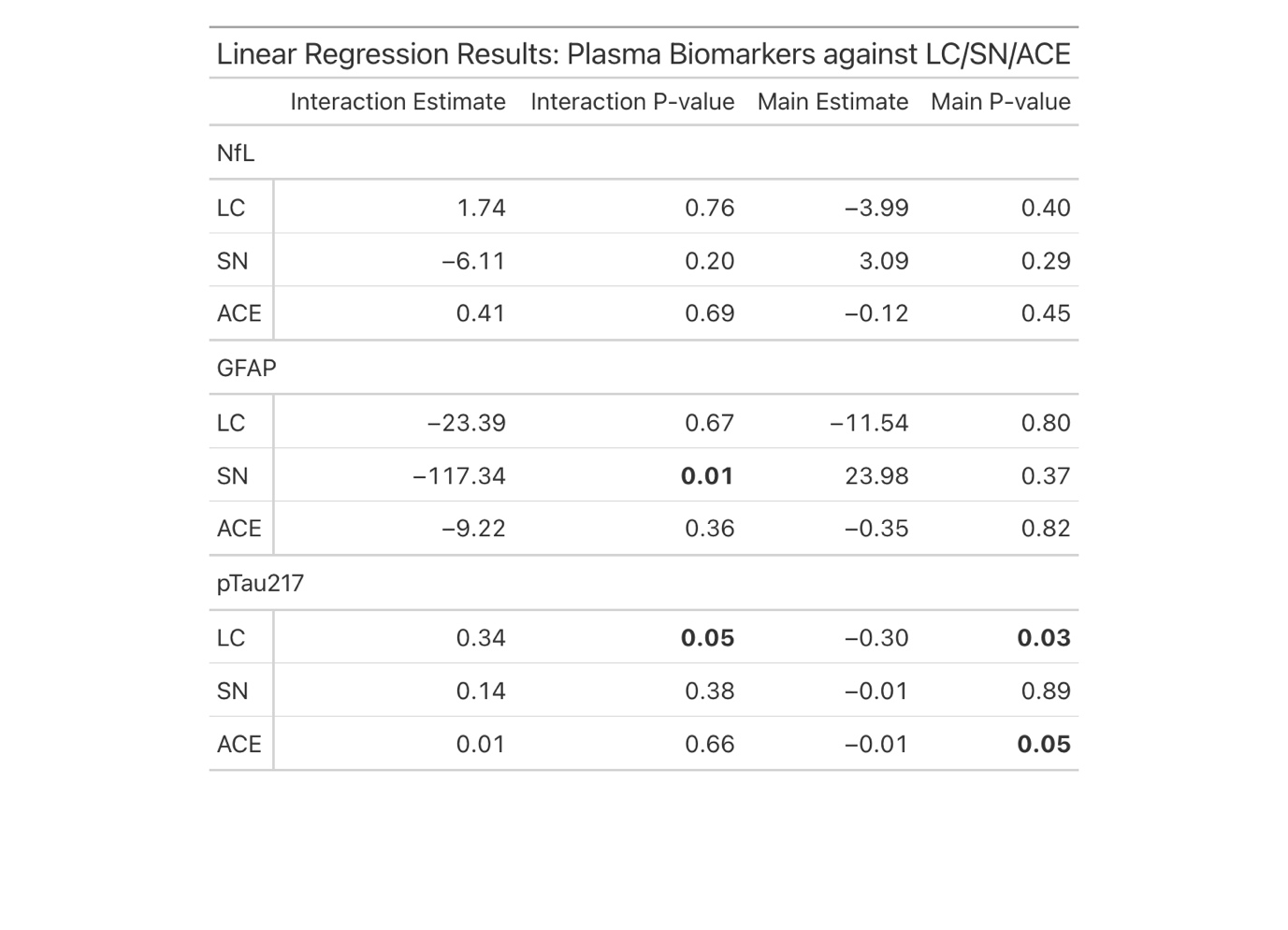
*

***Supplementary Table 4.*** *Table showing the results from linear regression models relating plasma biomarkers to LC/SN contrast and ACE. Each model run accounting for age, sex, and length of symptoms and with group as an interaction term, as well as education for ACE models. Significant P-values (<0.05) in bold. ACE=Addenbrooke’s Cognitive Examination; LC=locus coeruleus; SN=substantia nigra*
